# Integrated transcriptomic and metabolomic analysis reveals key pathways and metabolites underlying cold tolerance in passion fruit

**DOI:** 10.1093/hr/uhag164

**Published:** 2026-04-27

**Authors:** Lin-Hua Chen, Jiong Dong, Bing-Liang Fan, Bingrong Chen, Yongcai Huang, Liu Yang, Hao Guo, Wenguo Cai, Ling-Ling Chen

**Affiliations:** State Key Laboratory for Conservation and Utilization of Subtropical Agro-bioresources, College of Life Science and Technology, Guangxi University, Nanning 530004, China; State Key Laboratory for Conservation and Utilization of Subtropical Agro-bioresources, College of Life Science and Technology, Guangxi University, Nanning 530004, China; State Key Laboratory for Conservation and Utilization of Subtropical Agro-bioresources, College of Life Science and Technology, Guangxi University, Nanning 530004, China; State Key Laboratory for Conservation and Utilization of Subtropical Agro-bioresources, College of Life Science and Technology, Guangxi University, Nanning 530004, China; Crop Genetic Improvement and Biotechnology Laboratory, Guangxi Academy of Agricultural Sciences, Nanning 530007, China; Crop Genetic Improvement and Biotechnology Laboratory, Guangxi Academy of Agricultural Sciences, Nanning 530007, China; State Key Laboratory for Conservation and Utilization of Subtropical Agro-bioresources, College of Life Science and Technology, Guangxi University, Nanning 530004, China; State Key Laboratory for Conservation and Utilization of Subtropical Agro-bioresources, College of Life Science and Technology, Guangxi University, Nanning 530004, China; State Key Laboratory for Conservation and Utilization of Subtropical Agro-bioresources, College of Life Science and Technology, Guangxi University, Nanning 530004, China

## Abstract

Passion fruit (*Passiflora edulis*) production is significantly limited by sporadic cold stress events in unfavorable years. The existence of cultivars with varying cold tolerance offers opportunities for genetic improvement of this high-value crop. To elucidate the molecular basis of cold adaption in passion fruit, we conducted an integrated transcriptomic and metabolomic analysis comparing cold-tolerant and cold-sensitive passion fruit cultivars under low-temperature stress. Our multi-omics approach revealed that cold tolerance is associated with significant reprogramming of key pathways, particularly sphingolipid metabolism, phenylpropanoid metabolism, and terpene metabolism. We further validated that exogenous application of the key metabolites within these pathways, sphinganine, chlorogenic acid (CGA), rutin, and three terpenoids enhanced cold tolerance in cold-sensitive cultivars. Conversely, virus-induced gene silencing (VIGS) of core biosynthetic genes (e.g. *PesSPT*, *PesKDSR*, *PesHCT*, and *PesFG2*) in these pathways compromised cold resistance, leading to increased oxidative damage and more severe phenotypic injury. This study provides comprehensive insights into the molecular networks underlying cold tolerance in passion fruit, highlighting potential candidate genes and metabolites. Our findings establish a crucial foundation for future molecular breeding strategies aimed at improving cold resistance in this profitable horticultural crop.

## Introduction

Low-temperature stress poses a significant and growing threat to global agricultural output, with tropical crops such as passion fruit (*Passiflora edulis*) being exceptionally vulnerable due to their evolutionary origin in warm climates and consequent lack of innate freezing tolerance. The economic importance of this high-value crop continues to rise, as the fruit gains popularity worldwide, driving agricultural initiatives to extend its cultivation into cooler subtropical zones. However, as a thermophilic species, passion fruit is susceptible to chilling injury, which is a major hindrance to its cultivation in cooler regions [1]. Cold injury can manifest at temperatures below 8°C, and exposures approaching 0°C are frequently lethal, resulting in substantial yield loss [2]. Furthermore, even within traditional tropical growing areas, sporadic cold events can significantly reduce production [3, 4]. This escalating vulnerability highlights the critical necessity of enhancing the genetic and physiological resilience of passion fruit to temperature fluctuations.

While the molecular mechanisms of cold acclimation have been extensively characterized in model plants like *Arabidopsis thaliana*, which is centered on the conserved transcription regulatory module comprising C-repeat binding factors (CBFs), inducers of CBF expression (ICE), and the downstream cold-regulated (COR) genes [5, 6]. The transcriptome reprogramming further secures cells by stabilizing membranes, acting as osmoprotectants, and mitigating oxidative damage [7–9]. These well-defined signaling pathways are frequently inadequately developed in tropical species and their functions often exhibit significant divergence from those observed in temperate models. In passion fruit, prior investigations into cold tolerance have remained predominantly phenomenological, documenting physiological markers, such as malondialdehyde (MDA) accumulation, shifts in antioxidant enzyme activities, and osmotic adjustments [10–12]. Although these studies revealed varietal differences in cold resistance [13], which could be utilized to further elucidate the molecular mechanisms underlying this trait, they offered limited mechanistic insight [14]. Preliminary transcriptomic analyses hinted at the involvement of the ICE–CBF–COR pathway but were constrained by nascent genomic resources and did not explore broader metabolic reprogramming or validate causal genes [15, 16]. Consequently, a critical knowledge gap persists between observed physiological tolerance and a systems-level understanding of the regulatory and metabolic networks that confer cold adaptation in passion fruit.

We previously generated two high-quality passion fruit genome assemblies, which served as the reference genomes in this study [17]. Here, we performed an integrative analysis of transcriptomic and metabolomic profiles from cold-tolerant and cold-sensitive passion fruit cultivars subjected to controlled low-temperature stress. Our analysis revealed that cold tolerance is not merely a function of canonical stress pathways but involves a substantial reprogramming of several key metabolic pathways, most notably sphingolipid metabolism, phenylpropanoid metabolism, and terpene metabolism. We hypothesized that these pathways contribute critically to membrane stability, antioxidant capacity, and stress signaling. To move beyond correlation and establish causality, we functionally validated key players through a combination of exogenous metabolite application and virus-induced gene silencing (VIGS). Exogenous application of core metabolites, including sphinganine, chlorogenic acid (CGA), rutin, and terpenoids significantly enhanced cold tolerance in sensitive cultivars. Conversely, silencing of critical biosynthetic genes (*PesSPT*, *PesKDSR* in sphingolipid pathway; *PesHCT*, *PesFG2* in phenylpropanoid pathway) compromised cold resistance, leading to elevated oxidative damage and more severe phenotypic injury. Our findings transcend conventional pathway associations by pinpointing specific, validated genetic components and metabolic effectors for cold tolerance in passion fruit, thereby securing the sustainable cultivation of this economically important crop in the face of climatic variability.

## Results

### Metabolomic profiles of cold-tolerant and cold-sensitive passion fruits response to cold stress

**Figure 1:**
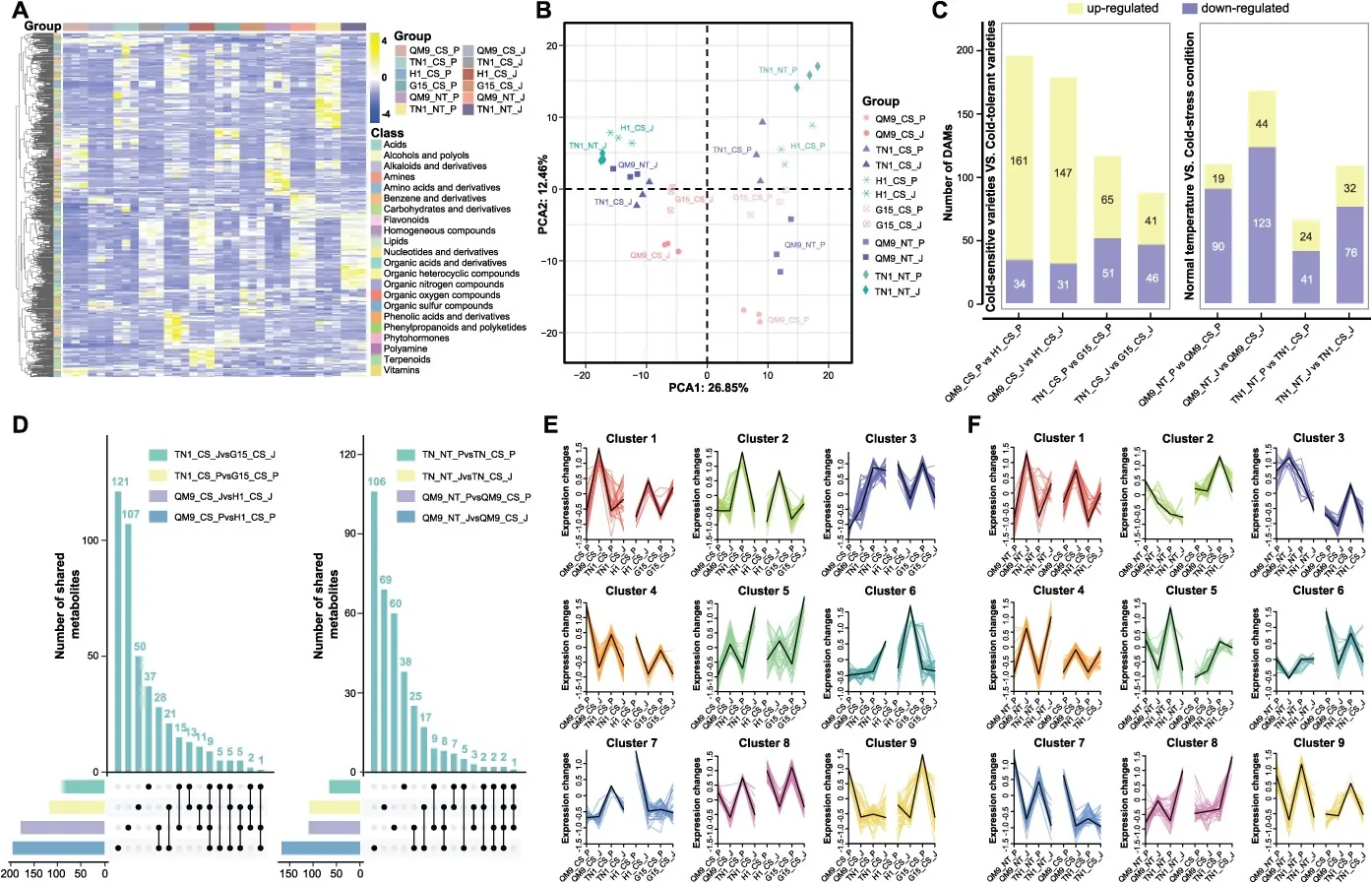
Comparation of the pericarp and pulp metabolomic profiles of cold-tolerant and cold-sensitive varieties. (A) Hierarchically clustered heatmap of 1236 metabolites from QM9, TN1, H1, and G15 passion fruits. The false color scale is depicted on the right side of the figure. (B) PCA of all metabolites generated in different tissues and genotypes. (C) Statistics of up- and downregulated DAMs in QM9 vs. H1, TN1 vs. G15, QM9_NT vs. QM9_CS, and H1_NT vs. H1_CS. (D) Advanced Venn diagram (UpSet) of the metabolome in these passion fruit species. The total number of DAMs in each time point is shown on the left. (E and F) K-means clustering of the nine clusters of metabolites between QM9 vs. H1, TN1 vs. G15 (E), and QM9_NT vs. QM9_CS, TN1_NT vs. TN1_CS (F), respectively. The *x*-axis depicts every group, and the *y*-axis depicts the *Z*-score standardized per metabolite.

In this study, we employed four species of passion fruit, two yellow passion fruit (*P. edulis* f. *flavicarpa*, YPF) and two purple passion fruit (*P. edulis* Sims, PPF). The YPF cultivar ‘Qinmi 9’ (QM9) and the PPF cultivar ‘Tainong 1’ (TN1) were identified as cold-sensitive, while the YPF cultivar ‘Dangling 1’ (H1) and the PPF cultivar ‘G15’ were clas sified as cold-tolerant. Stem, leaf, and fruit samples were collected from the cultivars QM9, TN1, H1, and G15 grown under natural conditions with ambient temperatures of 7°C–11°C (cold stage, CS). For the cold-sensitive varieties QM9 and TN1, corresponding samples were collected under normal temperatures (27°C–31°C, NT) as controls. To explore the differences in the cold-associated metabolic networks between cold-tolerant (H1 and G15) and cold-sensitive varieties (QM9 and TN1) under cold stress, fruit samples pericarp (P) and pulp (J) were subjected to a widely targeted metabolic and volatile organic compound profiling method by using liquid chromatography–mass spectrometry (LC–MS) and gas chromatography–mass spectrometry (GC–MS), respectively [18–21]. A total of 1236 metabolites were identified, including 15 acids, alcohols and polyols, 33 alkaloids, 28 amines, 203 amino acids and derivatives, 12 benzene and derivatives, 117 carbohydrates, 158 flavonoids, 116 lipids, 78 nucleotide and derivatives, 265 organic derivatives, 78 phenolic acids and derivatives, 43 phenylpropanoids and polyketides, 16 phytohormones, and 4 polyamine, 49 terpenoids and 21 vitamins (Supplementary Table S1). Analysis of differential metabolites revealed that only a small portion of the metabolites exhibited significant similarities in accumulation among the four passion fruit genotypes (Fig. 1A). These specific metabolites are likely contributed to the differentiation of cold-responsive metabolism between cold-tolerant and cold-sensitive passion fruits and their respective adaptation and tolerance mechanisms. Furthermore, principal component analysis (PCA) indicated clear segregation of the 1236 metabolites among four passion fruit varieties, which aligned with the results from hierarchical cluster analysis (Fig. 1B).

By comparing the metabolomic data from cold-tolerant and cold-sensitive passion fruits (QM9 vs. H1, TN1 vs. G15), we identified 334 differentially accumulated metabolites (DAMs) between QM9 vs. H1 and 178 DAMs between TN1 vs. G15 based on cutoff criteria of variable importance in the projection (VIP) ≥ 1 and |log2Fold Change| ≥ 1. Specifically, 161 upregulated and 34 downregulated metabolites were identified in the pericarp, and 147 upregulated and 31 downregulated metabolites were identified in the pulp of H1 vs. QM9 (Supplementary Fig. S1, Supplementary Table S2). In addition, in the comparison of TN1 vs. G15, 65 upregulated and 51 downregulated metabolites were detected in the pericarp of G15, and 41 upregulated and 46 downregulated metabolites were found in the pulp of G15 (Fig. 1C, Supplementary Table S2). When comparing metabolomic data from sensitive passion fruit at normal and cold temperature (NT vs. CS) for QM9 and TN1, we identified 238 DAMs (NT vs. CS) in QM9 and 164 DAMs in TN1. Among them, when QM9_NT was compared with QM9_CS, there were 19 upregulated metabolites and 90 downregulated metabolites in the pericarp of QM9_CS, whereas 44 upregulated metabolites and 123 downregulated metabolites were found in the pulp of QM9_CS. In addition, when TN1_NT was compared with TN1_CS, there were 24 upregulated and 41 downregulated metabolites in the pericarp of TN1_CS, whereas there were 32 upregulated and 76 downregulated metabolites in the pulp of TN1_CS (Supplementary Fig. S2, Supplementary Table S3). To compare metabolite accumulation across each species, we generated an advanced Venn diagram (UpSet plot) to illustrate the differential and common metabolites between cold-tolerant and cold-sensitive passion fruits (Fig. 1D). The results revealed a higher number of commonly accumulated metabolites within species, highlighting significant differences in metabolite accumulation patterns between species. Collectively, these findings indicate that QM9 vs. H1, as well as TN1 vs. G15, exhibit distinct metabolite accumulation patterns in response to low temperature, which may be linked to their contrasting for cold tolerance.

To better understand the metabolic difference between cold-tolerant and cold-sensitive varieties under cold stress, we employed a K-means clustering algorithm to categorize 430 DAMs (the union of 334 DAMs from QM9 vs. H1 and 178 DAMs from TN1 vs. G15) into nine distinct clusters (Fig. 1E, Supplementary Fig. S3A, Supplementary Table S4). Notably, we observed significant accumulation of metabolites in two specific clusters (clusters 5 and 8) in cold-tolerant varieties subjected to cold treatment, both in pericarp and pulp, compared to cold-sensitive varieties. These cold-induced metabolites predominantly included alcohols, sugars, nucleotides, and flavonoids, which are known for their roles in plant responses to abiotic stress, underscoring their importance as conserved stress-responsive metabolites. In contrast, in clusters 3, 6, 7, and 9, metabolites were accumulated in the pericarp and/or pulp of cold-tolerant varieties (Supplementary Fig. S3C), which comprised various lipids (including sphingolipids, lyso-phosphatidylcholine, tanshinlactone, cyasterone, and arachidic acid), alcohols and polyols (including eudesmoyl quinic acid and inositol), alkaloids (including quinolines, pyrrolidines, piperidines, isoquinolines, and indoles), phytohormones (including abscisic acid, salicylic acid, O-glucoside, and salicylic acid), terpenoids (including przewaquinone, valepotriate, (−)-borneol, ptaquiloside, beta-ionone, and hecogenin), numerous amino acids and their derivatives, as well as additional flavonoids and phenylpropanoids. These metabolites were specifically accumulated in the H1 and/or G15 varieties when exposed to cold treatment. Additionally, a total of 352 DAMs (the union of 238 DAMs from QM9_NT vs. QM9_CS and 164 DAMs from TN1_NT vs. TN1_CS) metabolites in cold-sensitive varieties under normal and cold temperature conditions were also categorized into nine distinct clusters (Fig. 1F, Supplementary Fig. S3B, Supplementary Table S4). In cold-sensitive varieties subjected to cold treatment, we observed significant accumulation of metabolites in three specific clusters (clusters 2, 6, and 8) in both the pericarp and pulp, compared to those under NT conditions (Fig. 1F, Supplementary Fig. S3D). These cold-induced metabolites predominantly included numerous amino acids and their derivatives, various flavonoids (such as (+)-catechin, dracorhodin, selgin C-hexoside, *etc*.), terpenoids (including elemol and diosgenin), as well as other differential metabolites similar to those observed between cold-tolerant and cold-sensitive varieties mentioned above.

Analysis of DAMs using Kyoto Encyclopedia of Genes and Genomes (KEGG) revealed that metabolic pathways enrichment between H1 vs. QM9 is greater than that between G15 vs. TN1 (Supplementary Fig. S4, Supplementary Table S5). Additionally, the number of DAMs between H1 vs. QM9 is significantly higher than that between G15 vs. TN1 (Fig. 1C). Among cold-sensitive varieties, the number of DAMs and pathway enrichment between NT vs. CS in QM9 exceeded those in TN1 (Supplementary Fig. S5, Supplementary Table S6). These findings suggests that YPF of cold-tolerant H1 and cold-sensitive QM9 exhibit more pronounced metabolic difference in response to cold tolerance compared to PPF (cold-tolerant G15 vs. cold-sensitive TN1). This indicates YPF varieties exhibit more significant metabolic changes in response to cold environment.

Specifically, the metabolic enrichment pathways shared between pericarp and pulp of DAMs in H1 vs. QM9 and G15 vs. TN1 include flavone and flavonol biosynthesis, biosynthesis of amino acids, phenylalanine, tyrosine, and tryptophan biosynthesis, phenylpropanoid biosynthesis, flavonoid biosynthesis, glycolysis/gluconeogenesis, fructose and mannose metabolism, and purine metabolism (Supplementary Fig. S4, Supplementary Table S5). Similarly, the metabolic enrichment pathways observed in the DAMs of QM9 and TN1 under both normal and cold conditions largely align with these pathways (Supplementary Fig. S5, Supplementary Table S6). These findings suggest that, beyond the well-known stress-responsive metabolic pathways (e.g. flavonoid pathway, phenylalanine metabolism, sugar and alcohol synthesis, and phenolic acid synthesis), cold-tolerant passion fruit exhibits a specific and significant enrichment of DAMs related to amino acid and nucleotide metabolism compared to cold-sensitive varieties. It was thus hypothesized that differences in the biosynthesis and metabolism of these metabolites conferred greater cold resistance to H1 and G15.

### Cold-tolerant and cold-sensitive passion fruits show distinct transcriptomic alterations in response to cold stress

**Figure 2:**
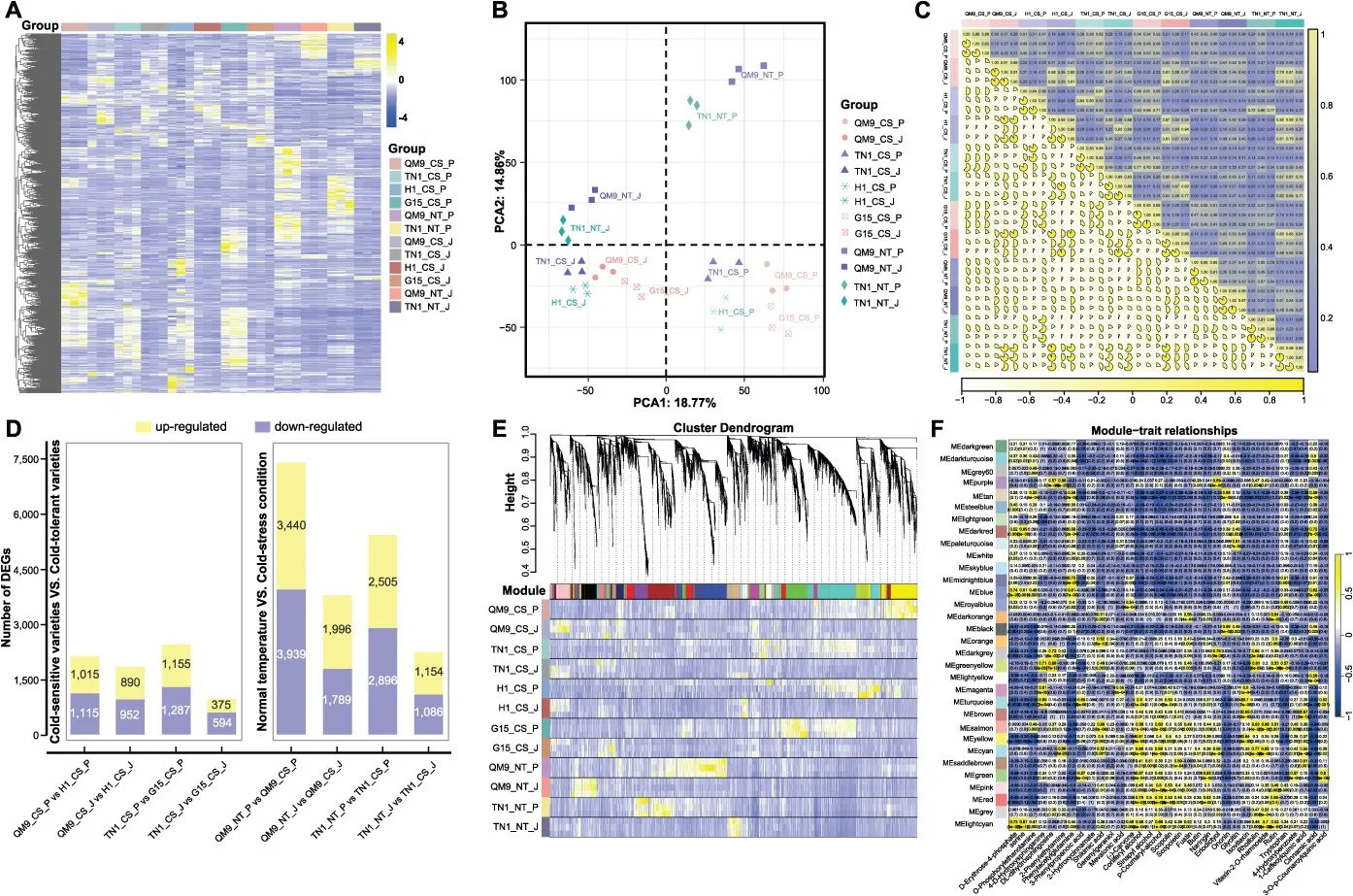
Comparation for the transcriptomic profiles of cold-tolerant H1 and cold-sensitive varieties. (A) Hierarchically clustered heatmap of gene transcripts from QM9, TN1, H1, and G15 passion fruits. The false color scale is depicted on the right side. (B) PCA of genes transcripts in different tissues and genotypes. (C) Hierarchically clustered heatmap of the gene transcripts. The completeness of pie chart corresponds to the magnitude of the correlation value. The false color scale is depicted on the right side. (D) Statistics of up- and downregulated DEGs in the QM9 vs. H1, TN1 vs. G15, QM9_NT vs. QM9_CS, and H1_NT vs. H1_CS. (E) Using weighted correlation network analysis (WGCNA), clusters of co-expressed genes were identified as modules. (F) Heatmap displays the relationship between these modules and compounds associated with cold response. The modules, represented by various colors, are aligned along the rows, while cold-related compounds are arranged along the columns.

Metabolic reprogramming is driven by transcriptional regulation; therefore, transcriptomic changes were further analyzed. To further investigate the molecular basis of transcriptomic adaptation to cold stress in cold-tolerant and cold-sensitive passion fruits, RNA-Seq data were generated using stem, leaf, and fruit (including pericarp and pulp) samples for transcriptomic analysis. Illumina HiSeq RNA sequencing generated an average of 7.35, 6.96, 6.36, 6.93, 7.07, and 6.83 Gb of high-quality reads for QM9_CS, TN1_CS, H1_CS, G15_CS, QM9_NT, and H1_NT, respectively, with an average mapping rate of 82.91%–95.73% (Supplementary Table S7) using our published yellow passion fruit genome as the reference [17]. Gene expression in all samples, with three biological replicates per tissue, was determined and provided in Supplementary Table S8. Heatmap profile showed consistent gene expression patterns within biological replicates of each tissue, while highlighting significant difference between cold-tolerant (H1 and G15) and cold-sensitive (QM9 and TN1) groups, as well as between cold condition groups (QM9_CS and TN1_CS) and the NT groups (QM9_NT and TN1_NT) of cold-sensitive varieties (Fig. 2A, Supplementary Fig. S6A). PCA of the gene expression data demonstrated that the first two principal components effectively distinguish all the samples, with PC1 and PC2 separating the samples based on the tissue types (including pericarp and pulp, stem and leaf) and genotypes, respectively (Fig. 2B, Supplementary Fig. S6B). Consistent with PCA results, correlation analysis revealed marked similarity among three biological replicates within each group, confirming the reliability and reproducibility of the transcriptomic data (Fig. 2C, Supplementary Fig. S6C). Taken together, these data suggested that gene expression profiles under cold treatment were also species-specific, consistent with the accumulation pattern of metabolites.

**Figure 3:**
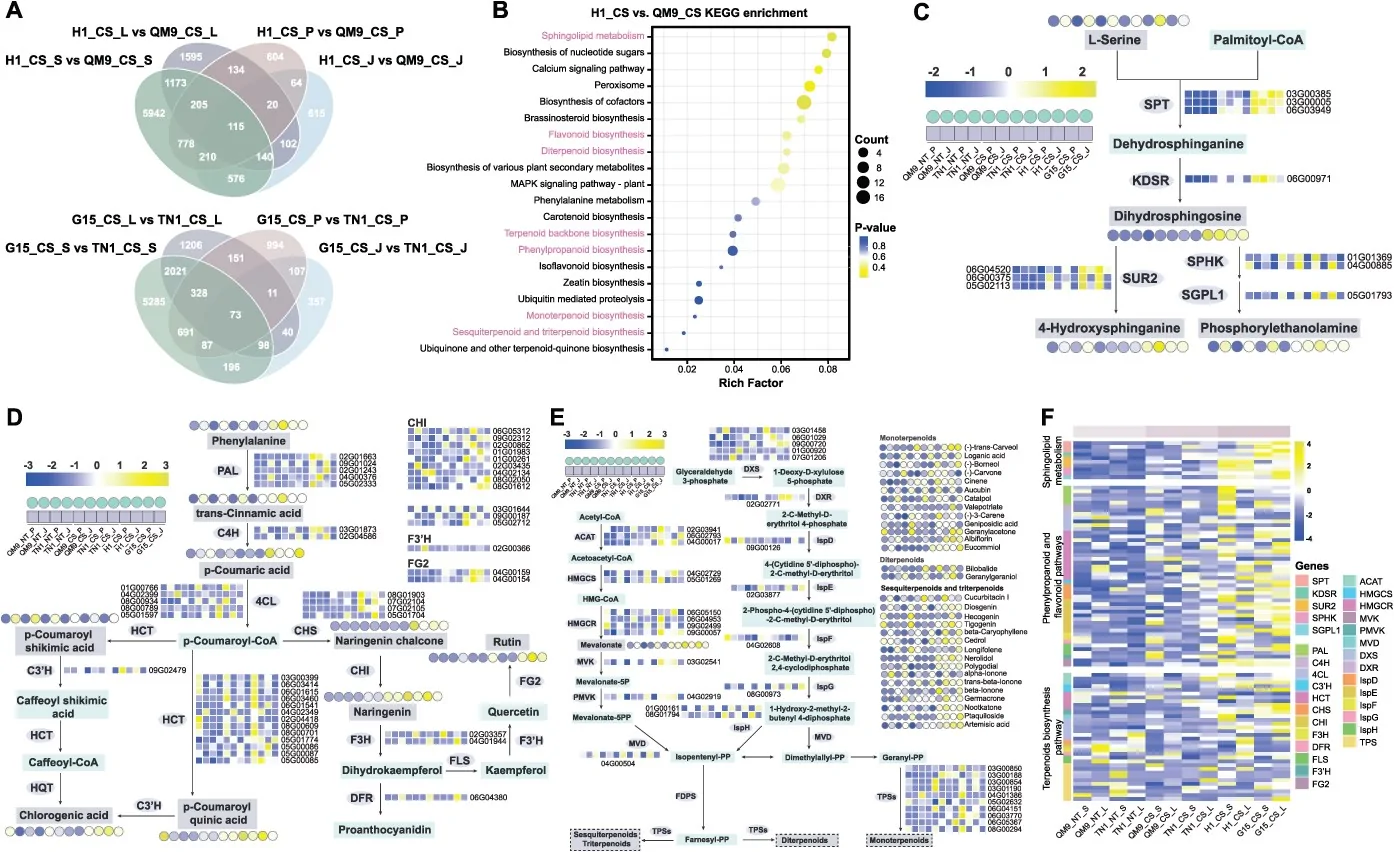
Key biosynthesis pathways in enhancing passion fruit cold tolerance. (A) Venn diagram analysis of common and specific genes between different pairwise comparisons. (B) The most enriched pathways among the species-dependent DEGs unique to H1 vs. QM9. (C) Metabolic pathway of SPH biosynthesis. The false color scale is depicted on the left side of the figure. Squares and circles represent gene expression and metabolite accumulation, respectively. (D) Expression profiles of CGA and rutin biosynthetic genes in passion fruits varieties. The false color scale is depicted on the left side of the figure. Squares and circles represent gene expression and metabolite accumulation, respectively (C3′H, 5-O-(4-coumaroyl)-D-quinate 3′-monooxygenase; DFR, dihydroflavonol 4-reductase). (E) Metabolic pathway of terpenoids biosynthesis. (F) Heatmap depicting gene expression in the biosynthesis pathways of SPH, CGA, and rutin in the stem (S) and leaf (L) of various passion fruit varieties.

Differentially expressed genes (DEGs) of these varieties were analyzed in cold-treated samples to gain further insights into the transcriptional changes under cold stress. Across four tissues, a total of 11 561 (5117 up- and 6444 downregulated) DEGs were identified in H1 vs. QM9, while a total of 10 741 (4905 upregulated and 5836 downregulated) DEGs were identified in G15 vs. TN1, using a threshold of adjusted *P*-value <0.05 and |log2Fold Change| > 1. Compared to QM9, the cold-tolerant variety H1 exhibited 1015 up- and 1115 downregulated DEGs in pericarp, 890 up and 952 downregulated DEGs in pulp, 4281 up- and 4858 downregulated DEGs in stem, and 1214 up- and 2270 downregulated DEGs in leaf (Fig. 2D, Supplementary Figs S6D, S7, and  S8, Supplementary Table S9). Similarly, in the comparison between G15 and TN1, the cold-tolerant variety G5 exhibited 1155 upregulated and 1287 downregulated DEGs in pericarp, 375 upregulated and 594 downregulated DEGs in pulp, 3982 upregulated and 4797 downregulated DEGs in stem, and 1803 upregulated and 2125 downregulated DEGs in leaf (Fig. 2D, Supplementary Figs S6D, S7, and  S8, Supplementary Table S9). When comparing gene expression in cold-sensitive varieties under normal and cold condition (QM9_NT vs. QM9_CS and TN1_NT vs. TN1_CS) across all four tissues, 13 576 DEGs (6203 upregulated and 7373 downregulated) were identified between QM9_NT and QM9_CS, and 12 016 DEGs (5365 upregulated and 6651 downregulated) between TN1_NT and TN1_CS. Specifically, in comparison of QM9_CS with QM9_NT, there were 3440 upregulated and 3939 downregulated DEGs in pericarp, 1996 upregulated and 1789 downregulated DEGs in pulp, 3847 upregulated and 4994 downregulated DEGs in stem, and 3009 upregulated and 3420 downregulated DEGs in leaf. Similarly, in the comparison of TN1_CS with TN1_NT, there were 2505 upregulated and 2896 downregulated DEGs in pericarp, 1154 upregulated and 1086 downregulated DEGs in pulp, 2927 upregulated and 3827 downregulated DEGs in stem, and 3009 upregulated and 3243 downregulated DEGs in leaf (Fig. 3D, Supplementary Figs S6D, S9, and  S10, Supplementary Table S10).

We further investigated the differences in transcriptional reprogramming of key metabolic pathways involved in cold stress adaptive responses in cold-tolerant and cold-sensitive varieties. Analysis of tissues-specific DEGs showed that there were 409 common DEGs in pericarp and pulp of H1 vs. QM9, and 1633 common DEGs in stem and leaf of H1 vs. QM9. Similarly, 278 common DEGs in pericarp and pulp of G15 vs. TN1, while 2520 common DEGs were found in stem and leaf of G15 vs. TN1 (Supplementary Figs S11A and S12A). KEGG pathway analysis revealed that these tissue-specific DEGs were mainly enriched in flavonoid metabolism (flavonoid biosynthesis, isoflavonoid biosynthesis, and flavone and flavonol biosynthesis), lipid metabolism (sphingolipid metabolism), phenylpropanoid metabolism (phenylalanine, tyrosine and tryptophan biosynthesis, phenylalanine metabolism, and phenylpropanoid biosynthesis), terpene metabolism (terpenoid backbone biosynthesis, monoterpenoid, diterpenoid, and sesquiterpenoid and triterpenoid biosynthesis), and mitogen-activated protein kinase (MAPK) signaling pathway across all tissues, for both H1 vs. QM9 and G15 vs. TN1 comparisons (Supplementary Figs S11B and S12B, Supplementary Table S11). Additionally, we analyzed cold-sensitive varieties under normal and cold conditions (QM9_CS vs. QM9_NT and TN1_CS vs. TN1_NT). The results aligned with the findings from cold-tolerant genotypes, showing similar pathways and metabolic processes (Supplementary Figs S13 and S14, Supplementary Table S12).

Gene Ontology (GO) annotation analysis revealed that the DEGs metabolic pathways between H1 vs. QM9 and G15 vs. TN1 were largely consistent in response to cold stress (Supplementary Fig. S15, Supplementary Table S13), with significant enrichment in carbohydrate metabolic processes (GO:0005975), signal transduction (GO:0007165), proteolysis (GO:0006508), methyltransferase activity (GO:0008168), lipid metabolic processes (GO:0006629), protein ubiquitination (GO:0016567), extracellular region (GO:0005576), and peroxisomes (GO:0005777). However, H1 vs. QM9 exhibited distinct enrichments in spliceosomal complex (GO:0005681), ubiquitin-protein transferase activity (GO:0004842), and regulation of gene expression (GO:0010468), whereas G15 vs. TN1 was specifically enriched in phosphorelay signaling (GO:0000160), oxidoreductase activity (GO:0016620), and transferase activity (GO:0016757). Furthermore, GO analysis of cold-sensitive varieties under normal and cold conditions revealed results generally consistent with those observed in cold-tolerant genotypes (Supplementary Fig. S16, Supplementary Table S14).

Weighted gene co-expression network analysis (WGCNA) was employed to further elucidate the regulatory mechanisms driving metabolic changes induced by cold stress in cold-tolerant and cold-sensitive varieties and explore co-expression networks. Based on gene expression similarities, 32 co-expression modules were identified (Fig. 2E, Supplementary Table S15). In the comparison of cold-tolerant (H1 and G15) vs. cold-sensitive (QM9 and TN1) varieties, genes predominantly expressed in the cold-tolerant varieties were clustered in turquoise, green, red, salmon, greenyellow, cyan, and lightcyan modules (Fig. 2E). Meanwhile, in the comparison between QM9 and TN1 under cold stress and NT, the genes preferentially expressed in QM9_CS and TN1_CS were primarily associated with yellow, magenta, turquoise, pink, and gray modules (Fig. 2E). The module–trait correlation heatmap (Fig. 2F, Supplementary Table S16) revealed strong associations between gene expression in lightcyan, magenta, turquoise, and greenyellow modules, and metabolites unique to H1 and G15 varieties. These metabolites included serine and sphingolipids (4-D-Hydroxysphinganine and DL-dihydrosphingosine). Moreover, genes within salmon, lightcyan, cyan, turquoise, green, red, greenyellow, and brown modules exhibited correlations with metabolites associated with coniferyl alcohol, *p*-coumaryl alcohol, naringin, 3-phenylpropanoic acid, 2-phenylethylamine, eriodictyol, rhoifolin, rutin, and other flavonoids and terpenoids, which were identified as the main conserved compounds involved in abiotic stress response. Therefore, our analysis highlighted a significant correlation between gene expression changes and metabolite production, suggesting these interactions play a crucial role in regulating cold stress response.

### Key pathways play a pivotal role in enhancing cold tolerance

To identify metabolic pathways potentially contributing to cold resistance in passion fruit, we performed KEGG pathway enrichment analysis on DEGs between cold-tolerant and cold-sensitive varieties. Specifically, we compared closely related genomic counterparts with contrasting cold resistance: H1 vs. QM9 and G15 vs. TN1. In total, 115 DEGs shared across stem, leaf, pericarp, and pulp in H1 vs. QM9, and 73 DEGs in G15 vs. TN1 were predominantly associated with stress-responsive pathways, including sphingolipid metabolism, MAPK signaling, flavonoid biosynthesis, terpene metabolism, and phenylpropanoid metabolism (Fig. 3A and B). Many components of the above pathways have been previously documented to function in cold tolerance for other plants, such as sphinganine in sphingolipid pathway for citrus [22], rutin and quercetin in flavonoid pathway for tomato seedlings and fruits [23, 24], and flavonoid pathways for rapeseed [25]. This indicates that our approach effectively identifies key metabolic pathways associated with the genetic basis of cold resistance variation among passion fruit varieties.

Sphingosine (SPH) metabolism is essential for synthesis and homeostasis for sphinglipid, which is a major plasmamembrane component and regulates cell membrane stability and fluidity, particularly under cold stress. Through WGCNA analysis, we identified 10 key genes in SPH metabolism within the turquoise co-expression module, including genes coding for serine palmitoyltransferase (SPT: *Pes03G0038500SH, Pes03G0000500SH*, and *Pes06G0394900SH*), 3-dehydrosphinganine reductase (KDSR: *Pes06G0097100SH*), sphinganine C4-monooxygenase (SUR2: *Pes06G0452000SH*), homologs of *SUR2* (*Pes06G0037500SH* and *Pes05G0211300SH*), sphingosine kinase (*SPHK*: *Pes04G0088500SH*), sphinganine-1-phosphate aldolase (*SGPL1*: *Pes05G0179300SH*). In addition, another co-expression module (yellow) included a gene coding for SPHK (*Pes01G0136900SH*). Specifically, *PesSPT*, *PesKDSR*, and *PesSUR2* exhibited higher expression levels in cold-tolerant varieties (H1 and G15) compared to cold-sensitive varieties (QM9 and TN1) (Fig. 3C–F). Additionally, homologs of *PesSUR2*, *PesSPHK*, and *PesSGPL1* showed higher expression levels in the pericarp of H1 and G15 than of QM9 and TN1 (Fig. 3C and F). These expression patterns suggest that genes involved in sphingolipid metabolism may collectively enhance cold tolerance of passion fruit.

Further investigation of phenylpropanoid and flavonoid pathways revealed CGA and rutin are important metabolites linked to cold responses in passion fruit (Fig. 3D). Key enzymes of the phenylpropanoid pathway, phenylalanine ammonia-lyase (PAL), cinnamic acid 4-hydroxylase (C4H), 4-coumarate-CoA ligase (4CL), and hydroxycinnamoyl-CoA shikimate/quinate hydroxycinnamoyl transferase (HCT/HQT), generate precursors for diverse metabolites, with HCT/HQT specifically directing CGA biosynthesis. Concurrently, the flavonoid pathway is governed by enzymes, including chalcone synthase (CHS), chalcone isomerase (CHI), flavanone 3-hydroxylase (F3H), flavonoid 3′-hydroxylase (F3′H), flavonoid 3′,5′-hydroxylase (F3′5′H), flavonol synthase (FLS), and flavonol-3-O-glucoside L-rhamnosyltransferase (FG2). Genes encoding *PAL*, *C4H*, *4CL*, *HCT/HQT*, *CHS*, *CHI*, *F3H*, *F3*′*H*, *FLS*, and *FG2* were significantly more induced under cold treatment in H1 and G15 varieties than in QM9 and TN1 (Fig. 3D and F). Previous studies have shown that exogenous application of compounds such as CGA enhance cold tolerance in tomato and *Citrus* [22, 23, 26], while applying rutin enhances broad stress resistance in rice and buckwheat [27, 28]. Our findings suggest that CGA and rutin, key metabolites we identified for cold resistance in passion fruit, may play a conserved role in cold resistance across plant species.

Moreover, our metabolomic data revealed that several terpenoids were detected in passion fruit, with higher accumulation observed in H1 and G15 compared to QM9 and TN1 (Fig. 3E). Consequently, we further analysis the terpene metabolic pathway. Meanwhile, in H1 and G15, the high expression of terpene synthase (*TPS*) genes was strongly correlated with the accumulation of multiple cold tolerance-related metabolites (Supplementary Fig. S17D and E), suggesting that these genes contribute to cold tolerance in passion fruit. The correlation between increased terpene production and the upregulation of *TPS* genes may further highlights the importance of terpene metabolism in the cold stress adaptive mechanisms of these cold-tolerant varieties.

### Validation of the candidate compounds and cold-responsive genes in passion fruit

**Figure 4:**
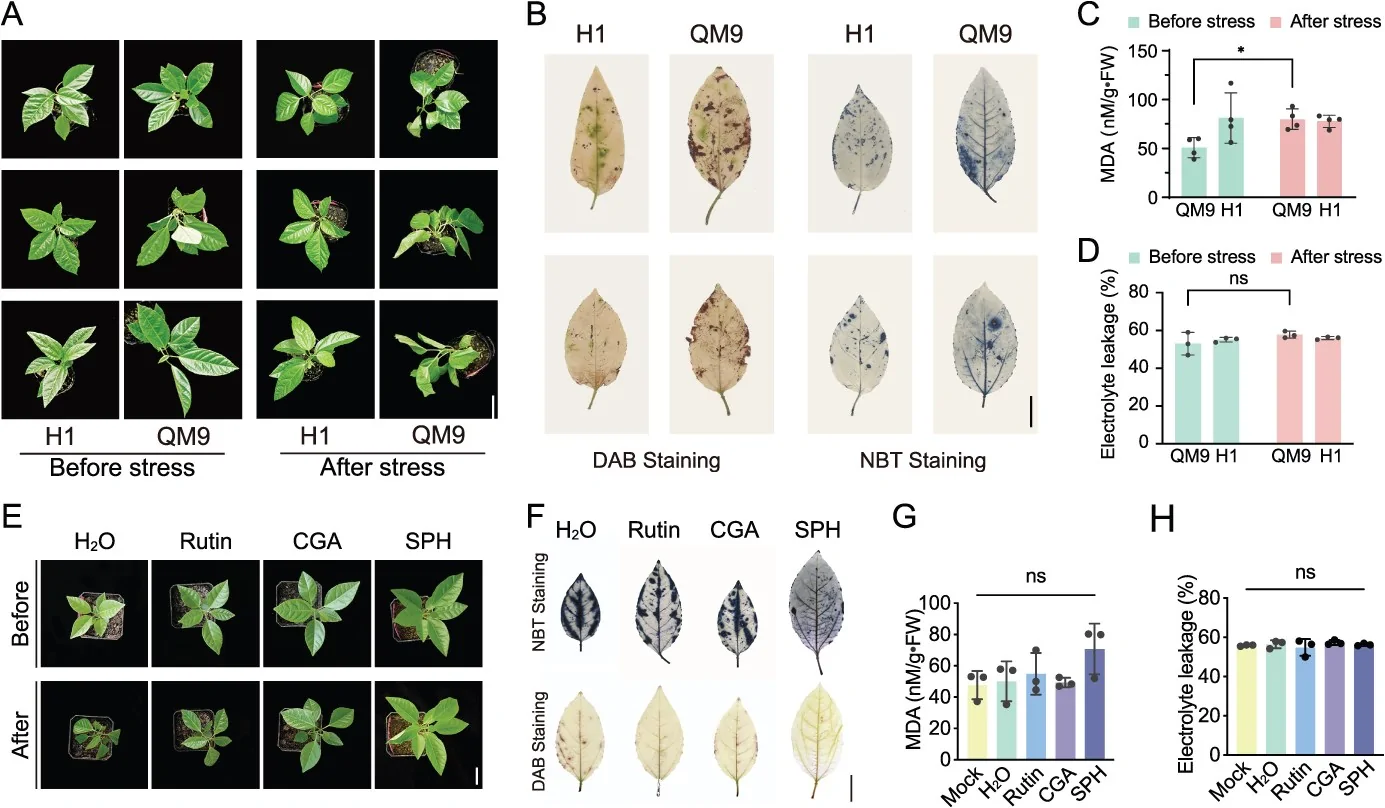
Phenotypic and physiological differences between cold-tolerant variety H1 and cold-sensitive QM9, and enhancement of QM9 cold tolerance by exogenous metabolite application. (A) Phenotypes of H1 and QM9 pre-and post-treatment (4°C, 6 hours). Scale bar, 5 cm. (B) DAB and NBT staining of H1 and QM9 leaves after cold treatment. Scale bar, 2 cm. (C and D) MDA content (C) and EL (D); ^*^P < 0.05; ns, no significant difference. (E) Phenotypic of QM9 pretreated with SPH, CGA, rutin and water before and after cold stress. Scale bar, 5 cm. (F) DAB and NBT staining of pretreated QM9 leaves under cold treatment. Scale bar, 2 cm. (G and H) MDA content (G) and EL (H) measured after cold treatment. Significant difference is determined by one-way ANOVA. ^*^P < 0.05; ^**^P < 0.01; ns, no significant difference. Data represent mean ± SD (*n* = 3). These experiments were repeated at least three times.

The difference in cold-tolerant variety H1 and cold-sensitive QM9 at seedling stage was validated with physiological parameters after cold treatment. We firstly evaluated the cold resistance phenotype of H1 and QM9, with their seedlings (2 months) exposed to cold treatment at 4°C for 6 hours. Under ambient temperature, no differences were observed between H1 and QM9 plants. After cold stress, more severe water-soaking and conspicuous leaf wilting were observed in QM9 in comparison to H1 (Fig. 4A). Under cold stress, plants typically accumulate excessive ROS, such as H₂O₂ and O₂^•–^. 3,3′-Diaminobenzidine (DAB) staining measuring H₂O₂ and nitroblue tetrazolium (NBT) staining measuring O₂•^−^ were used to examine oxidative damages. QM9 accumulated more reactive oxygen species (ROS) in the form of both H₂O₂ and O₂•^−^ than H1 (Fig. 4B). Since ROS further induces lipid peroxidation, MDA content was measured to indicate the oxidative damage in membranes. After cold treatment, MDA level accumulated significantly in QM9, whereas remains unchanged in H1 (Fig. 4C). Electrolyte leakage (EL), an indicator of membrane integrity, showed no significant differences between QM9 and H1 plants (Fig. 4D). Taken together, the above results indicate that H1 possesses superior cold tolerance to QM9 at the seedling stage.

Our transcriptomic and metabolomic analyses revealed significant enrichment of sphingolipid, phenylpropanoid, and flavonoid pathways in cold-tolerant passion fruit cultivars, particularly sphinganine, CGA, and rutin. We further conducted a hub-gene-centric network analysis. By extracting core genes such as *PesSPT* and *PesHCT* from the target modules, we reviewed their correlations with key metabolites of these pathways (Supplementary Fig. S18A and Supplementary Table S17). This integrative approach underscores a direct link between the transcriptional regulatory layer and the metabolic flux shifts essential for cold adaptation in passion fruit. To examine the cold protecting role of these chemicals, we performed exogenous spraying experiments on the cold-sensitive cultivar QM9. Leaf-spraying with sphinganine, CGA, and rutin alleviated leaf wilting under cold stress, treated-seedlings displaying markedly reduced damage compared with water sprayed mock controls (Fig. 4E, Supplementary Fig. S19A). DAB and NBT staining showed that sphinganine treatment significantly reduced ROS accumulation, whereas CGA and rutin treatments did not show a significant reduction (Fig. 4F). No significant difference in MDA content and EL was observed between treatment plant and control (Fig. 4G and H). Exogenous spraying sphinganine, CGA, and rutin thus enhanced cold tolerance in QM9, though their effects on oxidative damage varied, and exogenous spraying of CGA and rutin failed to increase the DPPH (2,2-diphenyl-1-picrylhydrazyl) radical scavenging activity in passion fruit leaves. (Supplementary Fig. S19B and C). Furthermore, three representative terpenoids, (−)-3-carene, nerolidol, and nootkatone were selected for exogenous spray experiments on the cold-sensitive cultivar QM9. The results demonstrated that the application of these compounds significantly enhanced the cold tolerance of QM9 (Supplementary Fig. S19D–G).

The regulation of key genes in the sphingolipid metabolism, phenylpropanoid metabolism, and terpene metabolism pathways involves a complex network of genes, including miRNAs and transcription factors (TFs). Among these, MYB TFs are particularly significant due to their essential role in regulating cold resistance-related genes. MYB TFs are a family of proteins that bind to specific DNA sequences to either activate or repress the expression of target genes. In our annotation of passion fruit, we identified over 700 TFs, including 170 MYB TFs (Supplementary Table S18). Analysis of the regulatory network revealed strong correlations between several key genes in the sphingolipid, phenylpropanoid, and terpene pathways and 371 stress-responsive TFs, encompassing families including MYB, EREBP-like factors, WRKY, bZIP, bHLH, HSF, MADS, and others (Supplementary Fig. S18B and Supplementary Table S19). Specifically, the homologous genes *pesSPT* (*Pes06G0394900SH*), *pesSUR2* (*Pes06G0452000SH*), *pesSGPL1* (*Pes05G0179300SH*), *pesHCT* (*Pes06G0161500SH* and *Pes04G0234900SH*), *pesFG2* (*Pes04G0015900SH*), *pesF3′H* (*Pes02G0036600SH*), and *pesTPS* genes (*Pes06G0415100SH*, *Pes06G0377000SH*, *Pes08G0029400SH*, *Pes03G0085000SH*, *Pes03G0085400SH*, and *Pes03G0119000SH*) exhibited strong correlations with various stress-related TFs, suggesting their crucial roles in the cold stress response.

**Figure 5:**
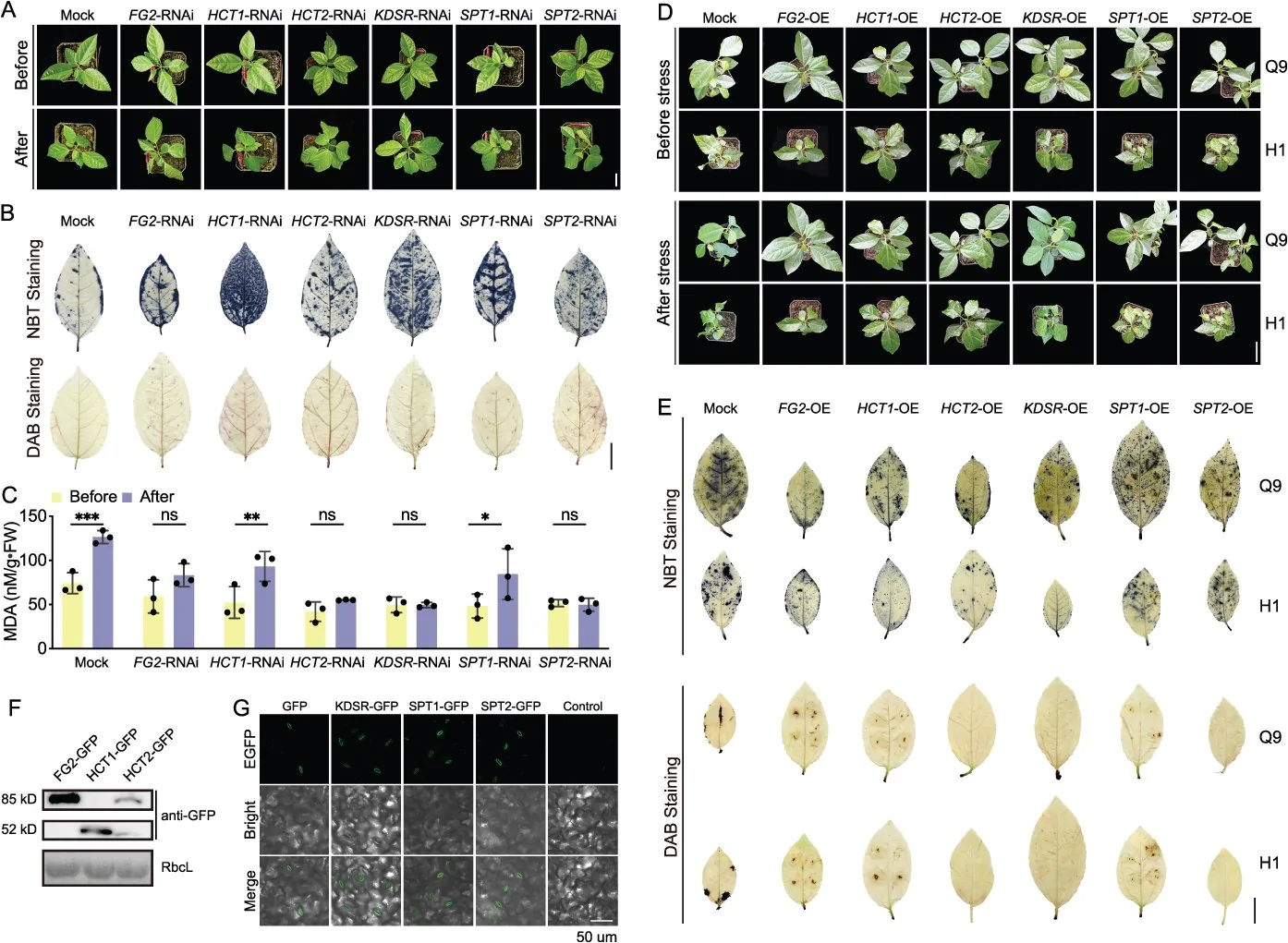
Silencing cold-tolerance genes weakened cold tolerance in H1, and overexpression of cold-tolerance genes enhanced the cold tolerance of QM9 and H1. (A) Phenotypes of VIGS-line H1 (silencing cold-tolerance-related genes) and the mock control plants (empty, TRV1 + TRV2) before and after cold treatment. Scale bar, 5 cm. (B) DAB and NBT staining of VIGS plants H1 and the mock control plants. Scale bar, 2 cm. (C) MDA content measured before and after cold treatment. (D) Phenotypes of over expression lines QM9, H1 (over expression cold-tolerance-related genes) and the mock control plants (empty vector, pXY104-GFP) before and after cold treatment. Scale bar, 5 cm. (E) DAB and NBT staining of OE plants QM9, H1 and the mock control plants. Scale bar, 2 cm. (F) Transient expression and protein detection of cold tolerance-related genes in passion fruit leaves. Target proteins fused with a GFP-tag were expressed in passion fruit leaves using an *Agrobacterium*-mediated transient transformation system. Representative bands from western blot analysis using an anti-GFP primary antibody are shown, confirming the successful expression of the target genes at the protein level. (G) Expression of GFP-fused protein observed under a laser scanning confocal microscope. Scale bar, 50 μm. Significant differences determined by two-way ANOVA in (C) (^*^P < 0.05, ^**^P < 0.01, ^***^P < 0.001; ns, no significant difference). Data represent mean ± SD (*n* = 3). These experiments were repeated at least three times.

Next, we sought to determine whether interrupting the endogenous biosynthetic pathways of sphinganine, CGA, and rutin would exacerbate cold damage. We employed tobacco rattle virus (TRV)-VIGS to silence (RNAi) sphingolipid biosynthesis-related genes *PesSPT* (*Pes03G0038500SH* and *Pes03G0000500SH*) and *PesKDSR* (*Pes06G0097100SH*), CGA biosynthesis-related genes *PesHCT* (*Pes04G0234900SH* and *Pes08G0060900SH*), and rutin biosynthesis-related gene *PesFG2* (*Pes04G0015411SH*). VIGS effectively silenced the above genes, which was validified with measuring expression levels of target genes with real-time quantitative PCR (RT-qPCR) (Supplementary Fig. S20A). Under ambient temperature, no obvious phenotypic difference was observed between plants with RNAi interfering target genes and the control plants (transfected with empty TRV1 and TRV2 vectors). Under cold treatment, VIGS plants displayed clearly more severe leaf wilting than the control plants (Fig. 5A, Supplementary Fig. S20B). In alignment, DAB and NBT staining revealed significantly higher ROS accumulation in VIGS plants compared with controls (Fig. 5B). Furthermore, MDA levels increased markedly in control plants after cold stress, whereas the increase was suppressed in VIGS plants, particularly in *PesFG2*-RNAi, *PesHCT2*-RNAi, *PesKDSR*-RNAi, and *PesSPT2*-RNAi plants, where MDA levels showed almost no increase under cold stress (Fig. 5C). To further validate the functional roles of these genes in cold tolerance, we constructed GFP-tagged overexpression vectors (pXY-104) for each candidate gene and performed *Agrobacterium*-mediated transient transformation in passion fruit leaves. Following cold treatment, overexpression of each gene significantly alleviated cold-induced leaf damage compared to the mock control, in both cold-sensitive and cold-tolerant varieties (Fig. 5D and E), and successful expression of the corresponding fusion proteins was confirmed by western blot analysis using an anti-GFP antibody and by fluorescence visualization under laser scanning confocal microscope (Fig. 5F and G). These results collectively support the positive role of these biosynthetic pathways in mitigating cold stress in passion fruit.

## Discussion

### Comprehensive metabolic reprogramming in cold-tolerant varieties

Our study demonstrates that cold-tolerant passion fruit varieties (H1 and G15) possess more complex metabolic regulatory network under cold stress compared to cold-sensitive varieties (QM9 and TN1). Through integrated metabolomic and transcriptomic analyses, we identified several key metabolic pathways and compounds that contribute to enhanced cold tolerance. Significant accumulation of various metabolites, including alcohols, polyols, sugars, lipids, alkaloids, terpenoids, amino acids, and flavonoids were observed in cold-tolerant varieties [9, 23, 29–31]. These compounds function as crucial osmoregulators, helping maintain intracellular water balance and prevent cellular dehydration under low-temperature conditions [32, 33]. Small-molecule sugars such as sucrose, glucose, and mannitol not only stabilize cell membrane structures but also provide essential energy, thereby improving plant cold tolerance [34–36]. Beyond these primary metabolites, H1 and G15 showed broader metabolic adjustments involving lipids, alkaloids, terpenoids, amino acids, and flavonoids, suggesting multilevel regulatory capacity in stress response [22, 37–39].

As major components of cell membranes, phospholipids play a vital role in maintaining membrane integrity and functionality under cold stress [40, 41]. The accumulation of dehydrosphinganine may help prevent physical damage caused by reduced membrane fluidity in low-temperature environments. Alkaloids and terpenoids, known for their antioxidant properties, enhance stress tolerance by modulating signaling pathways and eliminating excessive ROS. Similarly, flavonoids serve as important antioxidants, protecting cells from oxidative stress while participating in signaling and gene regulation processes. Additionally, we observed significant accumulation of nucleotides and phenylpropanoid metabolites in cold-tolerant varieties. Nucleotides support various stress response processes through their roles in energy metabolism and regulatory functions, while phenylpropanoid compounds contribute to antioxidant mechanisms and cell wall strengthening.

### Absence of ICE–CBF–COR pathway activation

Notably, our transcriptomic data did not reveal significant differential expression of the canonical ICE–CBF–COR pathway components in cold-tolerant passion fruit varieties. This conserved regulatory module, which serves as a cornerstone of cold acclimation in *Arabidopsis* and other plants [42–44], appears to play a minimal role in passion fruit’s cold adaptation. The absence of characteristic upregulation of ICE, CBF, and COR homologs suggests that passion fruit may have evolved alternative mechanisms for cold stress response, possibly relying more heavily on metabolic plasticity rather than this conserved transcriptional cascade. Previous studies have demonstrated that plant cold responses can occur through both CBF-dependent and CBF-independent regulatory pathways, indicating that not all cold-responsive genes are regulated through the ICE–CBF signaling cascade [43]. This finding underscores the species-specific nature of cold adaptation strategies and highlights the importance of not presuming universal conservation of established pathways across all plant species.

### Sphingolipid-mediated and ROS-independent cold protection

SPH accumulation was significantly higher in cold-tolerant passion fruit varieties, supported by elevated transcription of SPH synthesis genes, particularly *PesSPT*. Functional validation confirmed that exogenous SPH enhanced cold tolerance in sensitive varieties, while VIGS-mediated suppression of *PesSPT* reduced tolerance in resistant lines. Sphingolipids contribute to membrane integrity and fluidity under cold stress [45, 46], while sphinganine treatment notably reduced ROS accumulation, indicating additional roles in regulating antioxidant defenses. These results mirror observations in citrus, where SPH accumulation and the upregulation of related biosynthetic genes are essential for stabilizing plasma membranes and preventing EL under chilling stress [22].

CGA and rutin accumulation was markedly higher in tolerant varieties, with corresponding upregulation of biosynthetic genes *PesHCT* and *PesFG2*. Exogenous supplementation of CGA and rutin increases and RNA interruption of *PesHCT* and *PesFG2* decreases cold tolerance, respectively. Surprisingly, modulation of CGA and rutin did not change ROS level in our experiments, as both CGA and rutin accumulation are known antioxidants. In contrast, studies in tomato have shown that the accumulation of rutin and quercetin significantly enhances antioxidant capacity to alleviate low-temperature injury [23, 24]. However, in passion fruit, the effect of CGA and rutin on cold tolerance may be a consequence of alternative mechanisms, such as osmotic adjustment of secondary metabolism regulation. High performance liquid chromatography (HPLC) and DPPH radical scavenging assays demonstrated poor uptake of CGA and rutin by passion fruit under our experimental conditions. Exogenous application of these compounds failed to enhance the total antioxidant capacity of the leaves, suggesting that the protective role of CGA and rutin in passion fruit cold tolerance may be independent of the ROS-scavenging mechanism. Previous studies have reported that the suppression of specific secondary metabolic pathways can restructure the plant’s metabolic network through feedback mechanisms, potentially activating alternative ROS-scavenging systems (e.g. enhanced superoxide dismutase (SOD) or peroxidase (POD) activities) to maintain cellular redox homeostasis [47]. Furthermore, genetic modifications within the phenylpropanoid biosynthetic pathway have been demonstrated to induce complex transcriptional reprogramming; for instance, interfering with specific biosynthetic steps can activate transcriptional coactivators that coordinate cellular responses to maintain homeostasis [48]. These compensatory mechanisms may explain the observed suppression of oxidative damage following the silencing of *PesHCT* and *PesFG2* (Fig. 5G). Additionally, the potential pleiotropic effects of the VIGS system, arising from the presence of viral nucleotides and proteins, cannot be entirely ruled out. To minimize such interference, plants transfected with the empty vector (pTRV1 + pTRV2) were utilized as stringent controls to ensure that the observed phenotypic changes primarily resulted from the silencing of the target genes.

### Terpenoid diversification and defense

Cold-tolerant passion fruit varieties accumulated diverse terpenoids, including monoterpenes, diterpenes, and sesquiterpenes under cold stress, with concurrent upregulation of key terpenoid synthesis genes (*ACAT*, *MVK*, *DXR*, and TPSs). Some terpenoid compounds can also significantly improve the cold resistance of QM9 when applied exogenously. Terpenoids likely enhance cold tolerance through antioxidant activity, participation in phytohormone signaling, and membrane protection *via* lipid composition modification [37].

## Conclusion

In summary, our findings demonstrate that cold-tolerant passion fruit varieties employ a multifaceted metabolic strategy for cold adaptation, independent of the canonical ICE–CBF–COR pathway. The coordinated accumulation of protective metabolites across several biochemical pathways provides a robust defense system against low-temperature stress. These insights offer valuable theoretical foundations for developing strategies to enhance cold tolerance in passion fruit and other tropical crops through metabolic engineering or exogenous supplementation approaches.

## Materials and methods

### Plant materials and treatments

Seedlings of yellow passion fruit (*P. edulis* f. *flavicarpa*: QM9 and H1) and purple passion fruit (*P. edulis* Sims: TN1 and G15), collected from experimental fields at the Guangxi Academy of Agricultural Sciences, were transplanted into pots and grown under a 16-hour light/8-hour dark photoperiod at 25°C, followed by cold treatment (4°C for 6 hours) in a growth chamber. Additionally, under natural low-temperature conditions (7°C–11°C), leaves, stems, and fruits were collected from mature QM9, TN1, H1, and G15 plants in the experimental fields. For control groups, the same organs were collected from QM9 and TN1 plants under NT conditions (27°C–31°C). The fruits were further divided into pericarp and pulp for analysis.

### Metabolite extraction and profiling

Samples of pericarp and pulp from all passion fruit varieties were collected at the ripening stage to compare the metabolic differences between cold-tolerant and cold-sensitive varieties. Three biological replicates were utilized for the analysis of widely targeted metabolites and volatile organic compounds. The extraction procedure was as follows: (i) biological samples were freeze-dried using a vacuum freeze-dryer (Scientz-100F) and then ground with zirconia beads in a mixing mill (MM400, Retsch) at 30 Hz for 1.5 minutes; (ii) 100 mg of lyophilized powder was dissolved in 1.2 ml of 70% methanol solution, vortexed for 30 seconds every 30 minutes for a total of six cycles, and then stored in a refrigerator at 4°C overnight; (iii) after centrifugation at 12 000 rpm for 10 minutes, the extracts were filtered (SCAA-104, pore size 0.22 μm; ANPEL, Shanghai, China, http://www.anpel.com.cn/) prior to Ultra Performance Liquid Chromatography-Tandem Mass Spectrometry (UPLC–MS/MS) analysis. All metabolites were qualitatively analyzed using the MetWare database and quantitatively analyzed *via* multiple reaction monitoring mode. Metabolite data analysis was performed using Analyst 1.6.3 software. An Orthogonal Partial Least Squares Discriminant Analysis (OPLS-DA) model was established employing multiple supervised methods. Metabolites with VIP ≥ 1 and |log2Fold Change| ≥ 1 were considered DAMs. The KEGG compound database (http://www.kegg.jp/kegg/compound/) was utilized to annotate the identified metabolites, and the annotated metabolites were mapped to the KEGG pathway database (http://www.kegg.jp/kegg/pathway.html).

### Transcriptome sequencing and profiling

All samples were immediately frozen in liquid nitrogen after collection. Three biological replicates were conducted for each sample. RNA isolation, library construction, and sequencing were performed according to the RNA-seq analysis protocol for gene prediction. Raw reads were assessed for quality using FastQC, and duplicate sequences were removed with Trimmomatic, followed by mapping to the genome using Hisat2 [49, 50]. Transcripts were assembled using StringTie [51]. Gene expression was quantified by StringTie as fragments per kilobase of transcript per million fragments mapped. DEGs were identified using DESeq2 [52], with the false discovery rate applied to adjust the *P*-values. Genes exhibiting significant expression differences, with |log2Fold Change| ≥ 1 and adjusted *P*-values < 0.01, were considered DEGs and annotated with GO terms and KEGG pathways.

### Transcriptomic and metabolomic data analysis

PCA and hierarchical clustering were performed to compare gene and metabolite profiles in stems, leaves, pericarp, and pulp of passion fruit species. Data were normalized by *Z*-score transformation to ensure comparability across samples. The COMPLEXHEATMAP package in R was used for visualization. Enrichment analysis was performed based on the hypergeometric test, and pathway enrichment was analyzed using the KEGG database. Additionally, to explore co-expression relationships, unsigned co-expression networks were constructed using the WGCNA v1.69 package and visualized in Cytoscape.

### Physiological measurements

EL measurement: according to Zhong *et al.* [53], four leaf discs (0.5 cm diameter) were incubated in 20 ml deionized water at 25°C for 24 hours. Initial EL (EL_1_) was recorded, and samples were then heated at 95°C for 20 minutes, cooled to room temperature, and measured for final EL (EL_2_). The EL (%) was calculated as: EC_1_/(EC_1_ + EC_2_) × 100%.

MDA content measurement: MDA was measured using the thiobarbituric acid (TBA) method. Fresh leaves (0.1 g) were ground in liquid nitrogen, mixed with 5% trichloroacetic acid, and incubated on ice for 1 hour. After centrifugation, the supernatant was mixed with 0.6% TBA solution, and the mixture was heated at 95°C for 60 minutes. For the blank control, 200 μl of distilled water was used instead of sample supernatant. Absorbance at 532 and 600 nm was measured.

Hydrogen peroxide (H_2_O_2_) and superoxide anion (O_2_^•–^) detection: H_2_O_2_ accumulation was detected using DAB (CAS#: 91-95-2) staining and O_2_^•–^ accumulation was detected using nitrotetrazolium blue chloride (NBT, CAS#: 298-83-9) staining. Cold-treated leaves were incubated with either DAB or NBT solution at 37°C for 24 hours. After decolorizing in ethanol, the leaves were photographed [54].

The DPPH radical scavenging rate was calculated using a DPPH Free Radical Scavenging Capacity Assay Kit (Cat. No. BC4750; Beijing Solarbio Science & Technology Co., Ltd, China). After preparing the samples to be tested, the absorbance at 515 nm was measured. Ascorbic acid (10 mg/ml) was used as the positive control.

### LC–MS/MS analysis

Fresh leaves (0.1 g) were ground in liquid nitrogen, and 1 ml of 70% methanol was added for extraction. After vortexing and ultrasonic extraction, the samples were incubated at 4°C for 10–12 hours. The supernatant was collected and centrifuged at 14 000 rpm for 15 minutes at 4°C, and the final supernatant was used for LC–MS/MS analysis.

### Exogenous spraying experiment

Preliminary tests determined that the optimal spraying concentration of each compound was 10 mM. The solutions for spraying were prepared as follows: (i) 10 mM sphinganine: 100 mg D-erythro-dihydrosphingosine dissolved in 2 ml DMSO (dimethyl sulfoxide), diluted with 31.16 ml deionized water; (ii) 10 mM CGA: 106.293 mg dissolved in 1.5 ml DMSO, diluted with 28.5 ml deionized water; (iii) 10 mM rutin: 183.156 mg dissolved in 4 ml DMSO, diluted with 26 ml deionized water; (iv) 10 mM (−)-3-carene: 1 mg (−)-3-carene dissolved in 0.7341 ml of DMSO; (v) 10 mM nerolidol: 1 mg nerolidol dissolved in 0.4497 ml of DMSO; and (vi) 10 mM nootkatone: 1 mg of nootkatone dissolved in 0.458 ml of DMSO. At room temperature, QM9 and gene-silenced H1 seedlings were sprayed with water, 10 mM sphinganine, 10 mM CGA, or 10 mM rutin using a spray bottle. Each compound was applied twice at 24-hour intervals, and seedlings were subjected to cold treatment (4°C, 6 hours) 24 hours after the second application.

### VIGS and cold tolerance treatment

The VIGS system was established to target six key genes involved in sphingolipid, phenylpropanoid, and terpene metabolism: *FG2*, *HCT1*, *HCT2*, *KDSR*, *SPT1*, and *SPT2*. Gene-specific fragments were amplified and inserted into pTRV2 vectors (Supplementary Table S20), screened on kanamycin, verified by PCR and sequencing, and then introduced into *Agrobacterium tumefaciens* strain GV3101.

The *Agrobacterium* carrying pTRV1, pTRV2, or recombinant pTRV2 were grown in YEP medium at 28°C, collected by centrifugation, and resuspended in infiltration buffer (10 mM MES, 10 mM MgCl₂, and 200 μM acetosyringone). The cell density was adjusted to an OD_600_ of ~1.0 and incubated in the dark for 4 hours. Equal volumes of *Agrobacterium* containing pTRV1 and pTRV2 (or recombinant constructs) were mixed and infiltrated into 1-month-old H1 seedlings. Plants treated with pTRV1 + pTRV2 served as negative controls, while untreated plants served as mock controls. Each plant received 2 ml of the mixture in three target leaves. After 24-hour dark incubation at 25°C, seedlings were transferred to 25°C, 50% humidity, under a 16-hour light/8-hour dark cycle for 2 days. Samples were collected to assess silencing efficiency.

### RT-qPCR analysis

Total RNA was extracted from H1 seedlings using TRIzol (Labled, Beijing, China) reagent, and cDNA was synthesized using the Labled First-Strand cDNA Synthesis Mix kit. Quantitative PCR was performed using gene-specific primers (Supplementary Table S21) and a reference gene primer set from Wu *et al.* [55]. Relative expression levels of target genes were calculated using the 2^–ΔΔCt^ method.

### Statistical analysis

This study involved multiple repetitions of all experiments, with a minimum of two trials and three biological replicates per condition. Results are expressed as mean ± standard deviation (SD). Statistical evaluations were conducted using one-way analysis of variance (ANOVA) *via* SPSS (IBM, NY, USA) along with *t*-tests. Significance levels were established with ^*^P < 0.05, ^**^P < 0.01, and ^***^P < 0.001, reflecting statistically significant differences among the groups.

## Supplementary Material

Web_Material_uhag164

## Data Availability

The datasets generated and analyzed during this current study are included in this published article and its supplementary materials. The raw RNA-seq data have been deposited in the NCBI Sequence Read Archive (SRA) under the BioProject accession number PRJNA1415482. The corresponding BioSample data are available under the accession numbers SAMN54936929 to SAMN54936970. Furthermore, the metabolomics datasets have been deposited in the MetaboLights repository under the accession numbers MTBLS13980 (targeted metabolomics) and MTBLS13982 (GC–MS metabolomics).
