# Supplementary material for: Integrated transcriptomic and metabolomic analysis reveals key pathways and metabolites underlying cold tolerance in passion fruit
Source: Hortic Res. 2026 Apr 27;13(9):uhag164. doi: 10.1093/hr/uhag164 (PMC13523861; doi:10.1093/hr/uhag164)
Supplement: Supplementary file 1 [file web_material_uhag164.zip › Supplementary Figures-2.pdf]

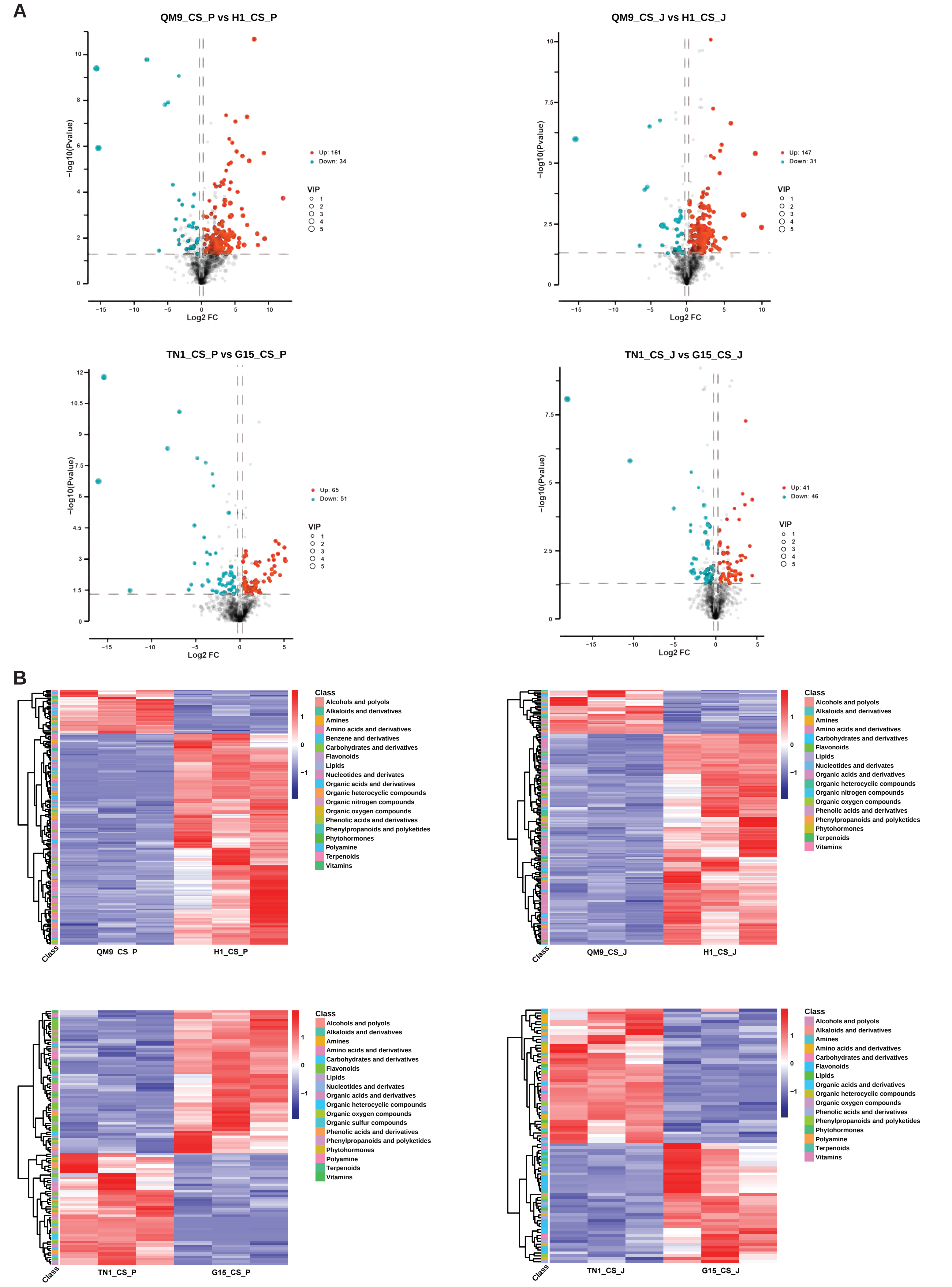

Supplemental Figure S1. Comparison of metabolic variation of pericarp (P) and pulp (J) between cold-tolerant and cold-sensitive passion fruits. A. Volcano plot of up- and down-regulated differentially accumulated metabolites (DAMs) in QM9 vs. H1, TN1 vs. G15. **B.** Heatmap of up- and down-regulated DAMs in QM9 vs. H1, TN1 vs. G15. P and J are the abbreviations for pericarp and pulp respectively; CS is the abbreviation for cold stage.

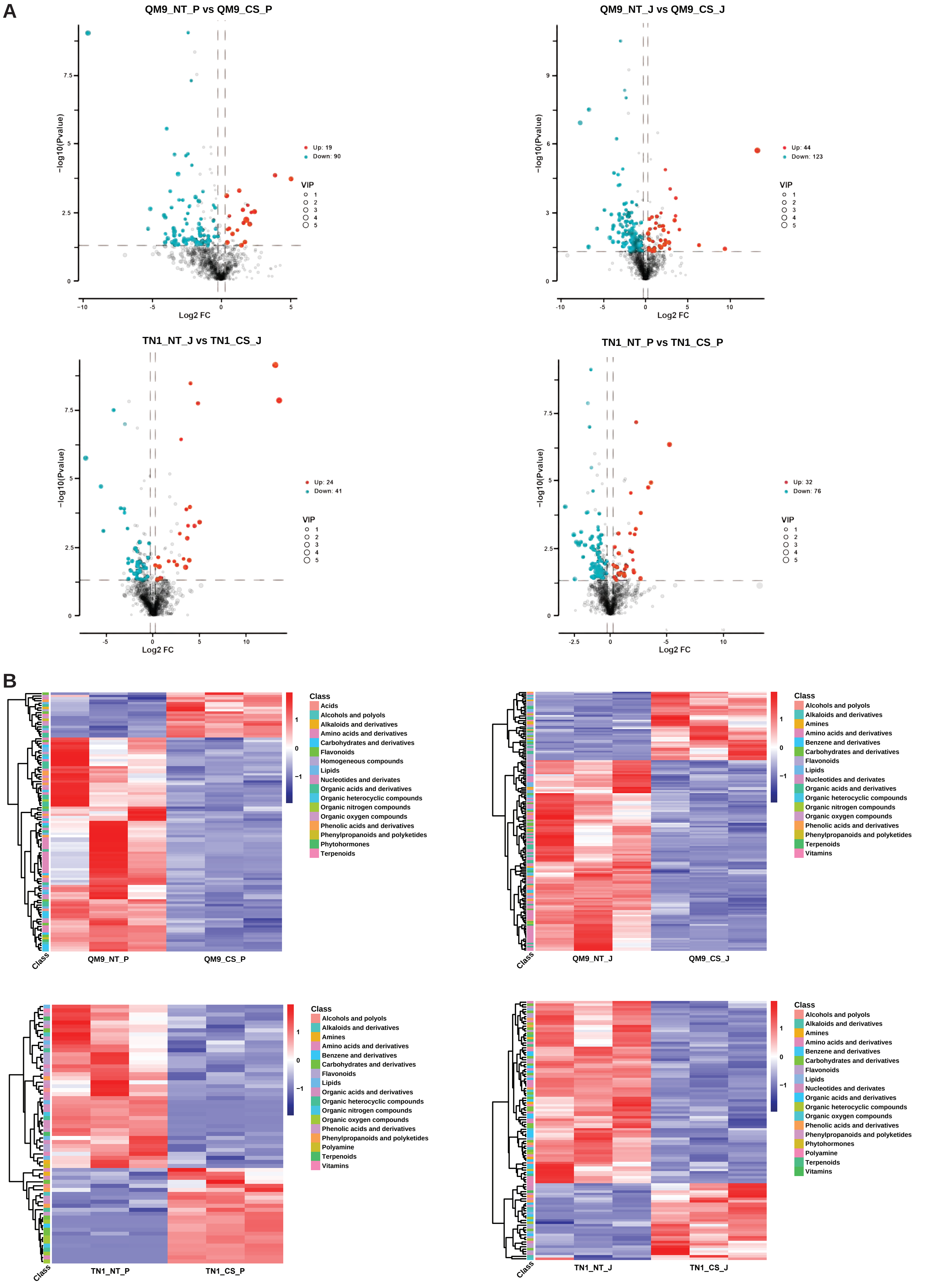

Supplemental Figure S2. Comparison of metabolic variation of pericarp (P) and pulp (J) of cold-sensitive passion fruits between cold stage and normal temperature. A. Volcano plot of up- and down-regulated differentially accumulated metabolites (DAMs) in QM9_NT vs. QM9_CS, TN1_NT vs. TN1_CS. **B.** Heatmap of up- and down-regulated DAMs in QM9_NT vs. QM9_CS, TN1_NT vs. TN1_NT. P and J are the abbreviations for pericarp and pulp respectively; NT and CS are the abbreviation for normal temperature and cold stage.

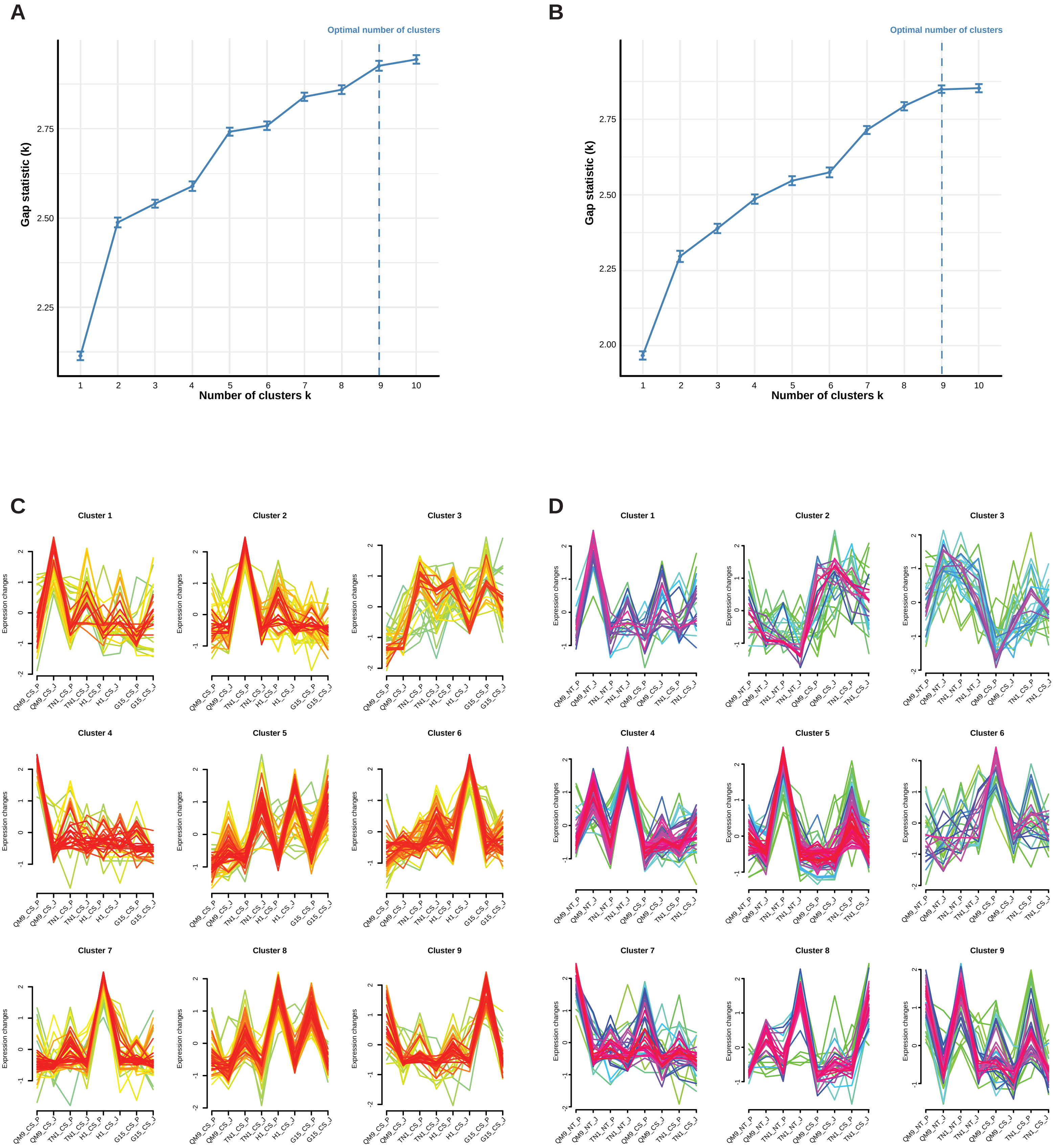

Supplemental Figure S3. Determination of the Optimal Number of Clusters Using the Gap Statistic. A and **B.** the gap statistic for different numbers of clusters (k) in k-means clustering of H1/G15 vs. QM9/TN1 (**A**), and QM9_CS/TN1_CS vs. QM9_NT/QM9_NT (**B**). The highest gap statistic of both are observed at k = 9, indicated by the dashed line, suggesting that 9 is the optimal number of clusters for this data set. **C** and **D.** K-means clustering of the 9 clusters of metabolites between QM9 vs. H1, TN1 vs. G15 (**C**), and QM9_NT vs. QM9_CS, TN1_NT vs. TN1_CS (**D**), respectively. The X-axis depicts every group, and the Y-axis depicts the Z-score standardized per metabolite. P and J are the abbreviations for pericarp and pulp respectively; NT and CS are the abbreviation for normal temperature and cold stage.

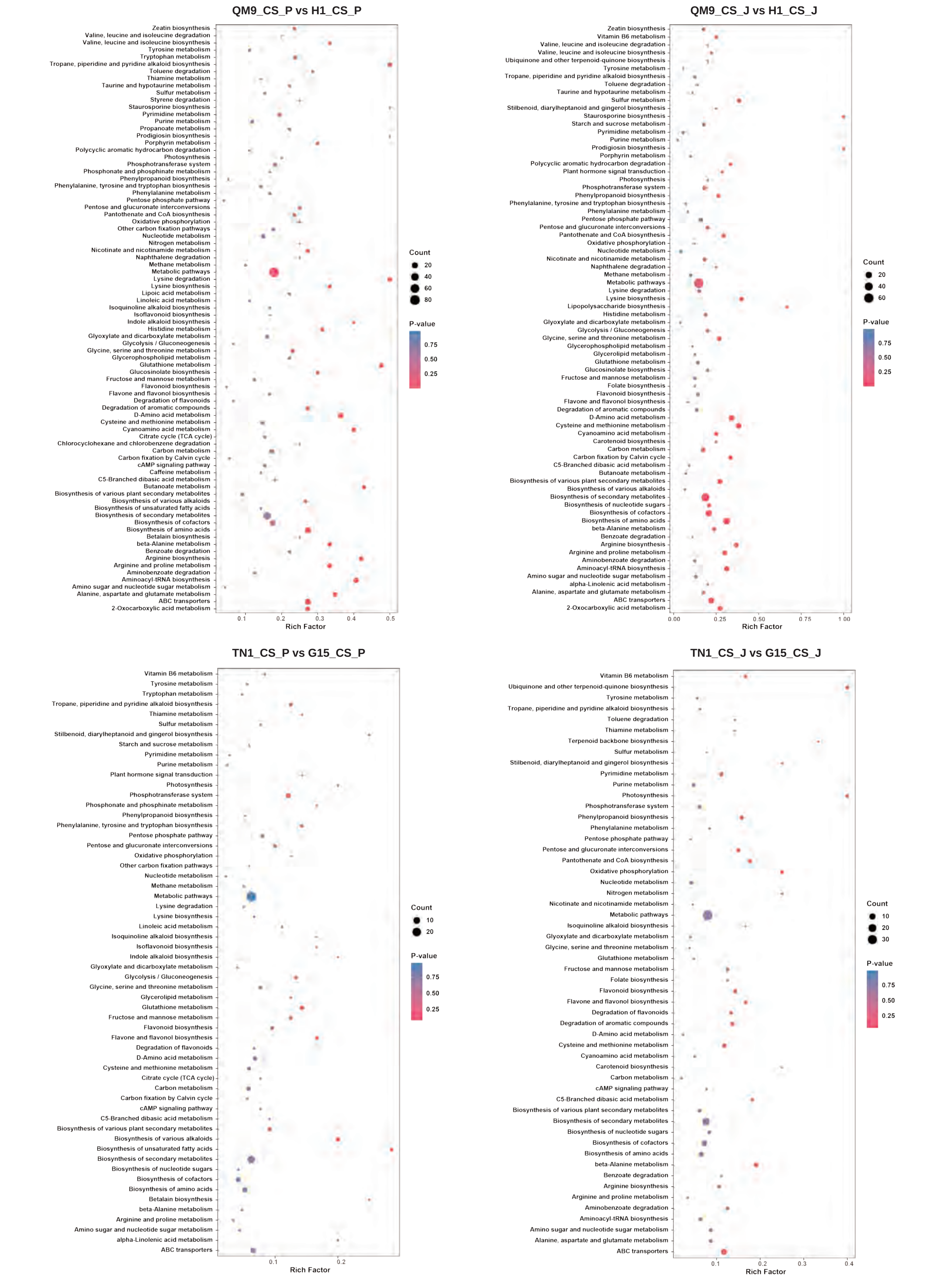

Supplementary Figure S4. The KEGG enrichment analysis of DAMs between cold-tolerant and cold-sensitive passion fruits. The KEGG enrichment analysis of DAMs from cold-tolerant (H1 and G15) compared to cold-sensitive (QM9 and TN1) varieties in cold treatment. Circle color denotes the P-value, circle size is proportional to the number of DAMs involved in the pathway (Count).

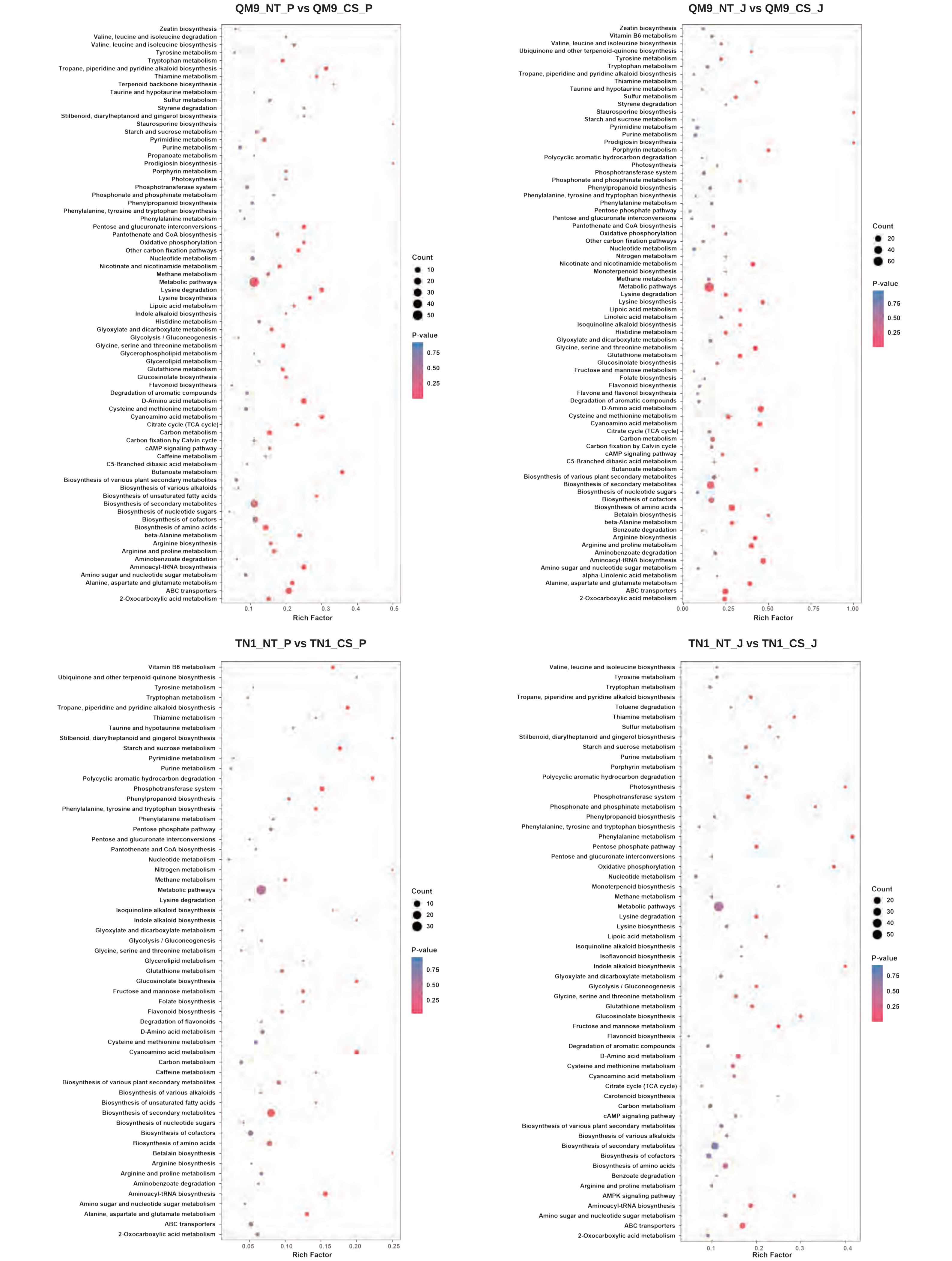

Supplementary Figure S5. The KEGG enrichment analysis of DAMs of cold-sensitive passion fruits between cold stage and normal temperature. The KEGG enrichment analysis of DAMs between QM9_NT vs. QM9_CS, and TN1_NT vs. TN1_CS. Circle color denotes the P-value, circle size is proportional to the number of DAMs involved in the pathway (Count).

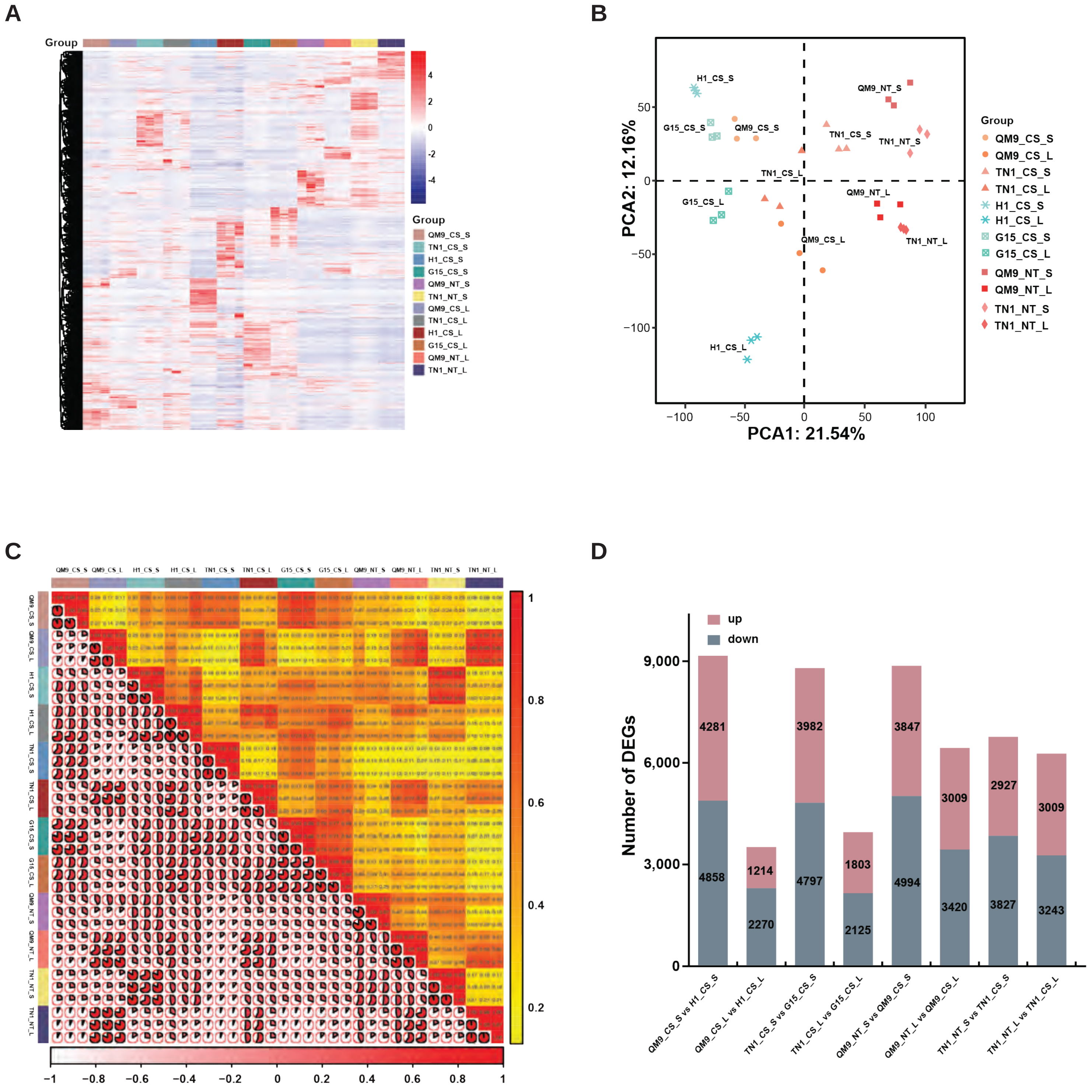

Supplemental Figure S6. Transcriptomic alterations of stem (S) and leaf (L) of passion fruit varieties. A Hierarchically clustered heatmap of gene transcripts from QM9, TN1, H1 and G15 passion fruits. The false color scale is depicted on the right side of the figure. **B** Principal component analysis (PCA) of genes transcripts in different tissues and genotypes. **C** Hierarchically clustered heatmap of the gene transcripts. The completeness of the pie chart corresponds to the magnitude of the correlation value. The false color scale is depicted on the right side of the figure. **D** Statistics of up- and down-regulated differentially expressed genes (DEGs) in the QM9 vs. H1, TN1 vs. G15, QM9_NT vs. QM9_CS, and H1_NT vs. H1_CS.

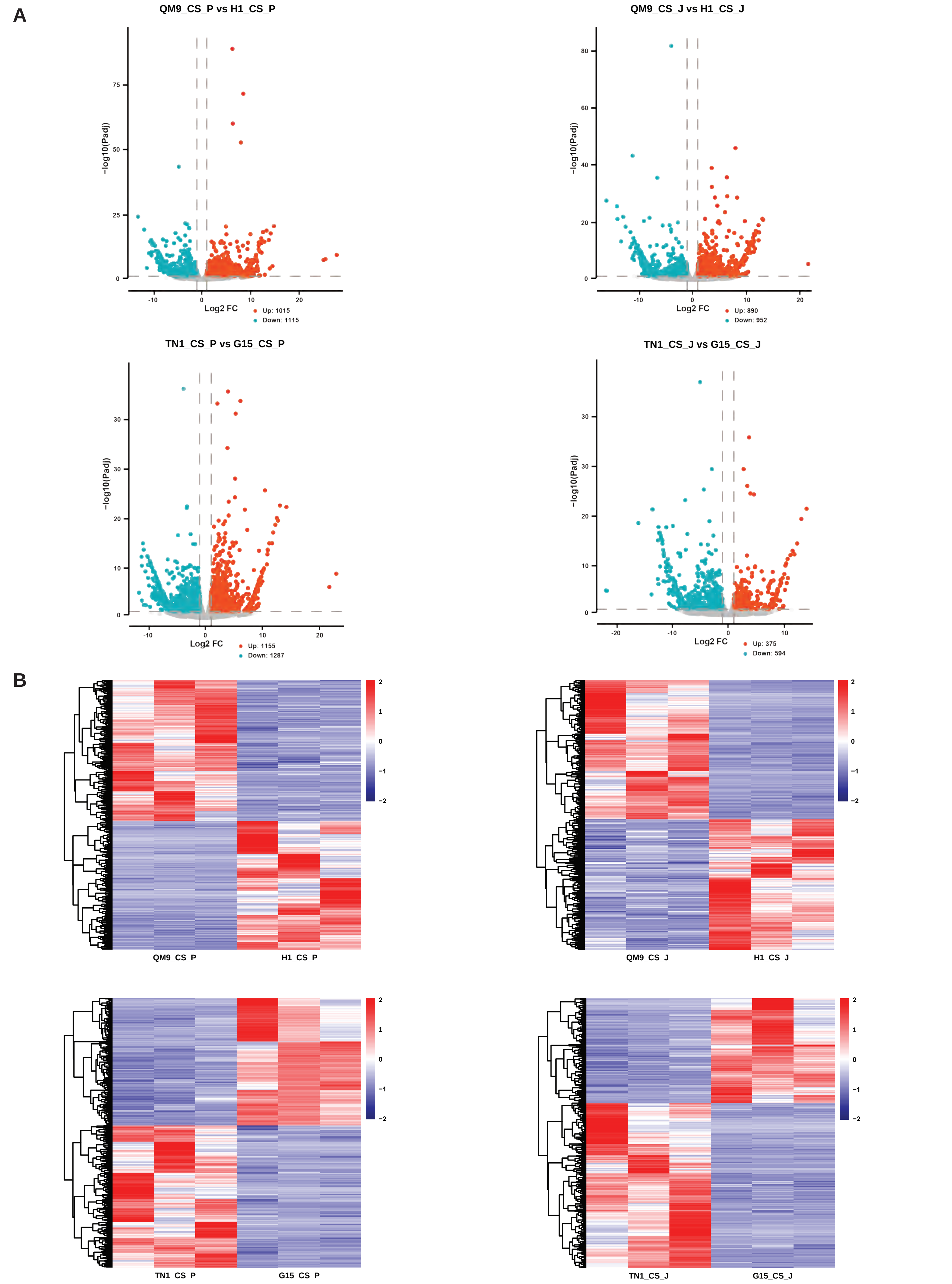

Supplemental Figure S7. Comparison of transcriptomic alterations of pericarp (P) and pulp (J) between cold-tolerant and cold-sensitive passion fruits. A. Volcano plot of up- and down-regulated differentially expressed genes (DEGs) in QM9 vs. H1, TN1 vs. G15. **B.** Heatmap of up- and down-regulated DEGs in QM9 vs. H1, TN1 vs. G15. P and J are the abbreviations for pericarp and pulp respectively; CS is the abbreviation for cold stage.

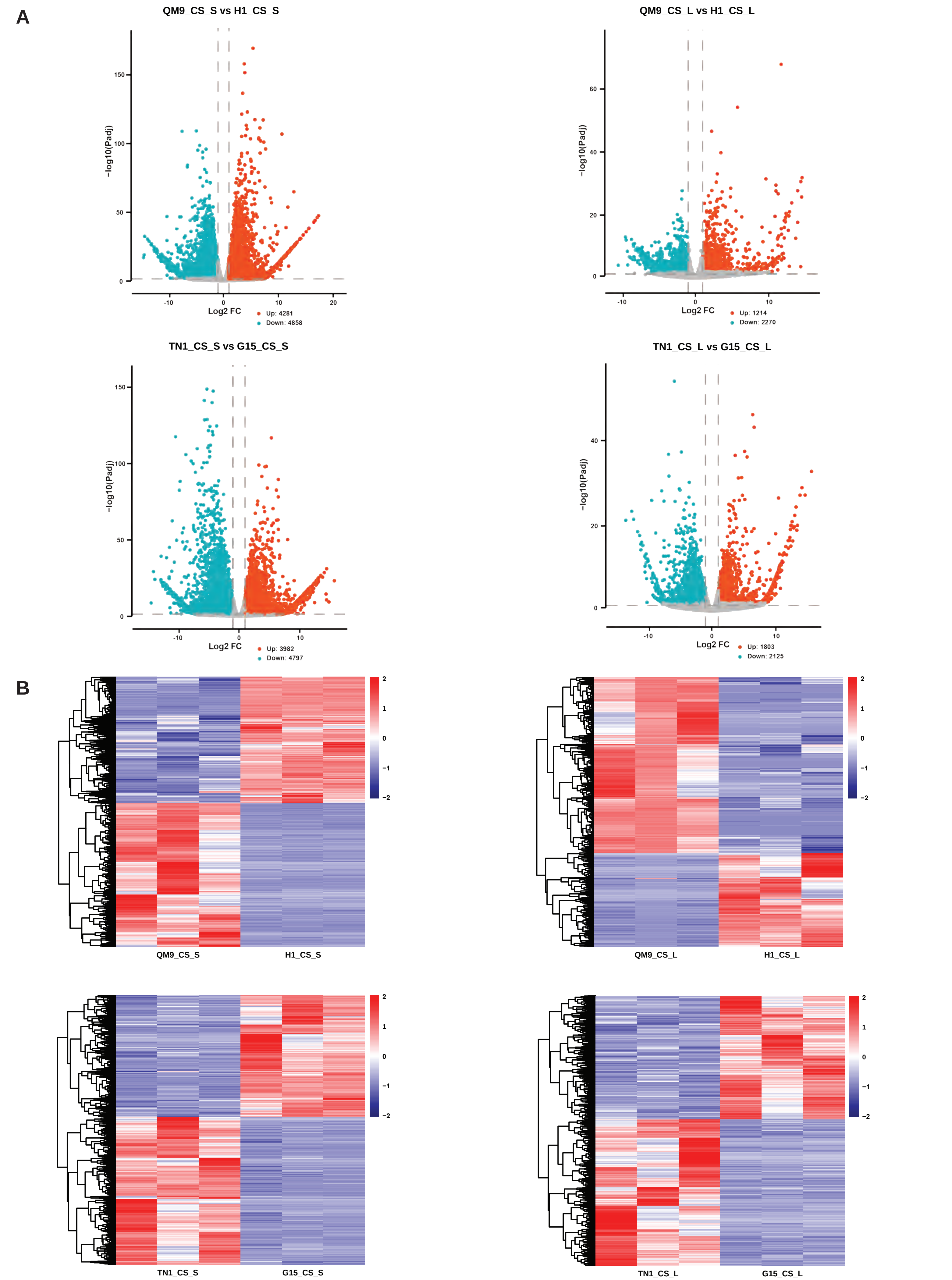

Supplemental Figure S8. Comparison of transcriptomic alterations of stem (S) and leaf (L) between cold-tolerant and cold-sensitive passion fruits. A. Volcano plot of up- and down-regulated differentially expressed genes (DEGs) in QM9 vs. H1, TN1 vs. G15. **B.** Heatmap of up- and down-regulated DEGs in QM9 vs. H1, TN1 vs. G15. S and L are the abbreviations for stem and leaf respectively; CS is the abbreviation for cold stage.

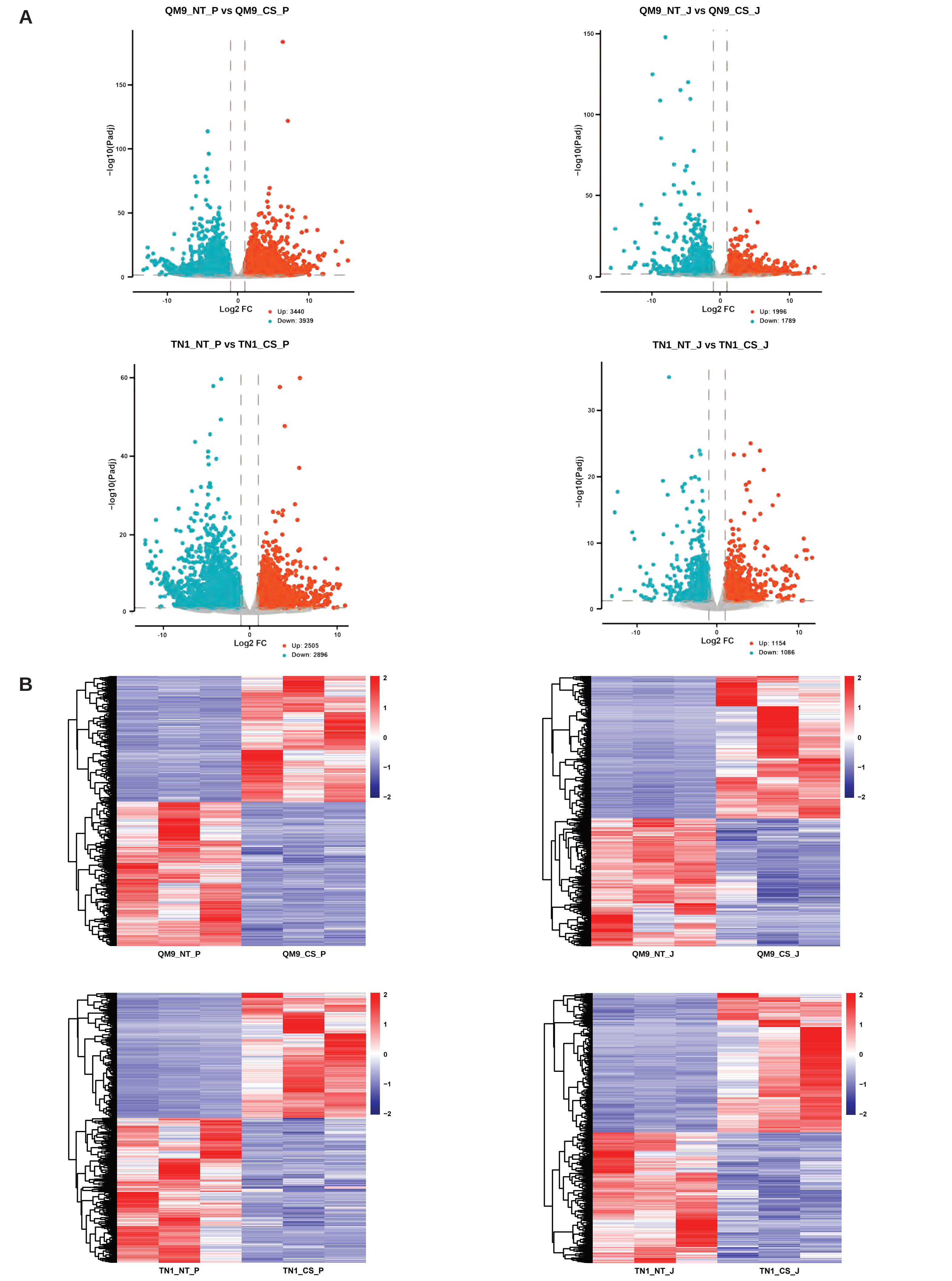

Supplemental Figure S9. Comparison of transcriptomic alterations of pericarp (P) and pulp (J) of cold-sensitive passion fruits between cold stage and normal temperature. A. Volcano plot of up- and down-regulated differentially expressed genes (DEGs) in QM9_NT vs. QM9_CS, TN1_NT vs. TN1_CS. **B.** Heatmap of up- and down-regulated DEGs in QM9_NT vs. QM9_CS, TN1_NT vs. TN1_NT. P and J are the abbreviations for pericarp and pulp respectively; NT and CS are the abbreviation for normal temperature and cold stage.

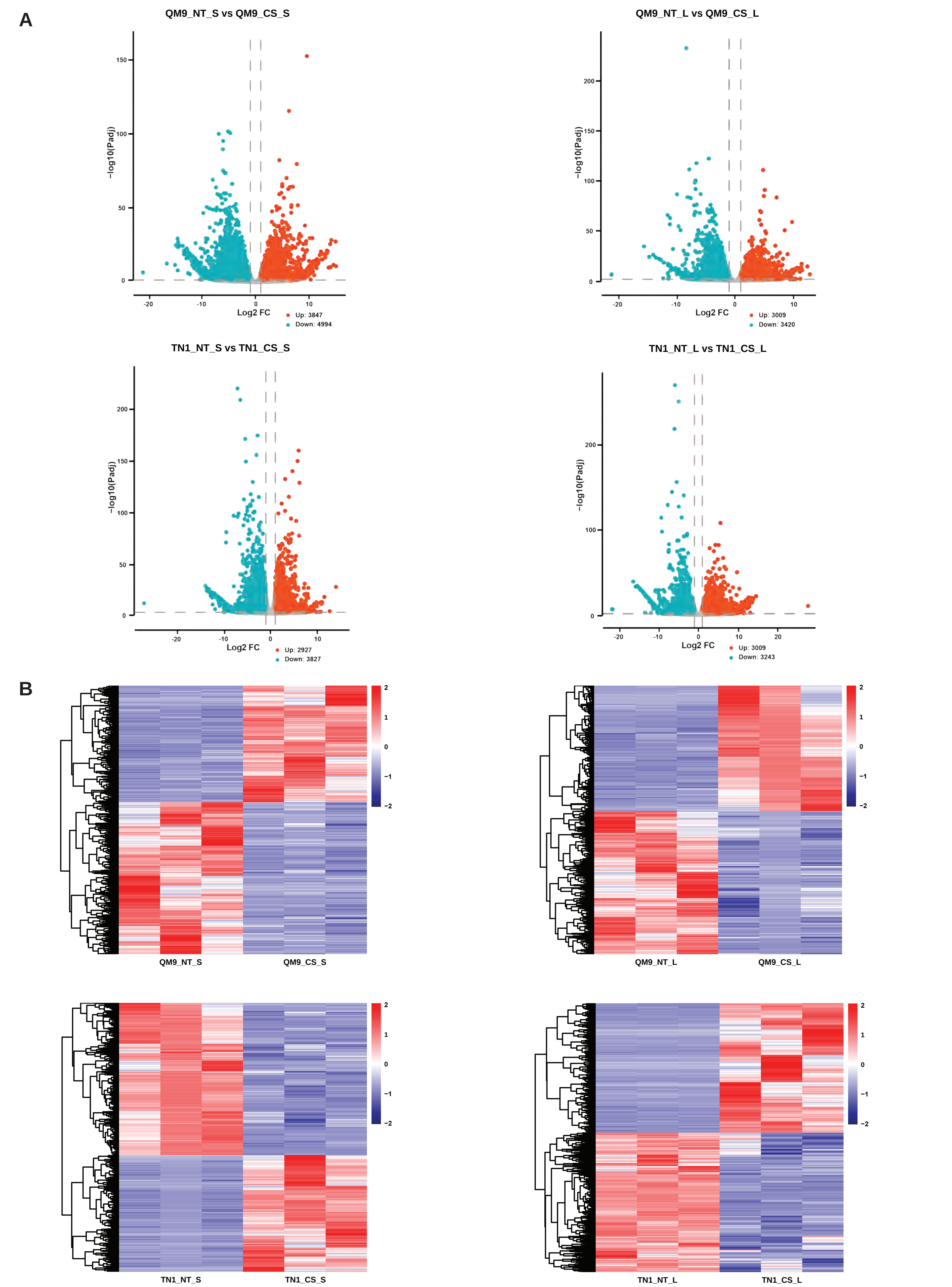

Supplemental Figure S10. Comparison of transcriptomic alterations of stem (S) and leaf (L) of cold-sensitive passion fruits between cold stage and normal temperature. A. Volcano plot of up- and down-regulated differentially expressed genes (DEGs) in QM9_NT vs. QM9_CS, TN1_NT vs. TN1_CS. **B.** Heatmap of up- and down-regulated DEGs in QM9_NT vs. QM9_CS, TN1_NT vs. TN1_NT. S and L are the abbreviations for stem and leaf respectively; NT and CS are the abbreviation for normal temperature and cold stage.

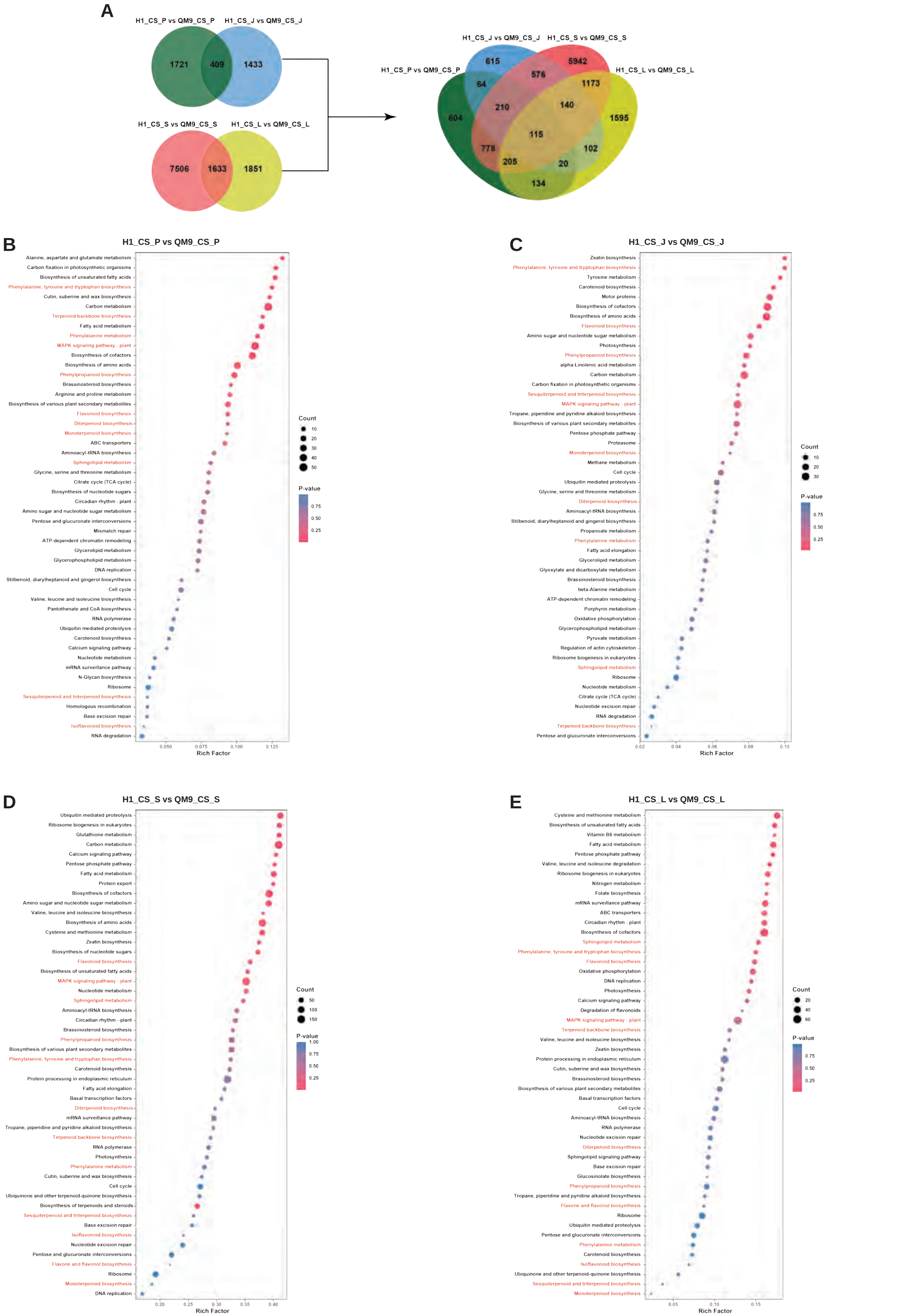

Supplementary Figure S11. The KEGG enrichment analysis of DEGs between H1 and QM9 passion fruits. A. Venn diagram analysis of common and specific genes between different pairwise comparisons. **B-E.** KEGG enrichment analysis of peels (P), pulp (J), stem (S) and leaf (L) of DEGs from H1 compared to QM9 varieties in cold treatment, respectively. Circle color denotes the P-value, circle size is proportional to the number of DAMs involved in the pathway (Count).

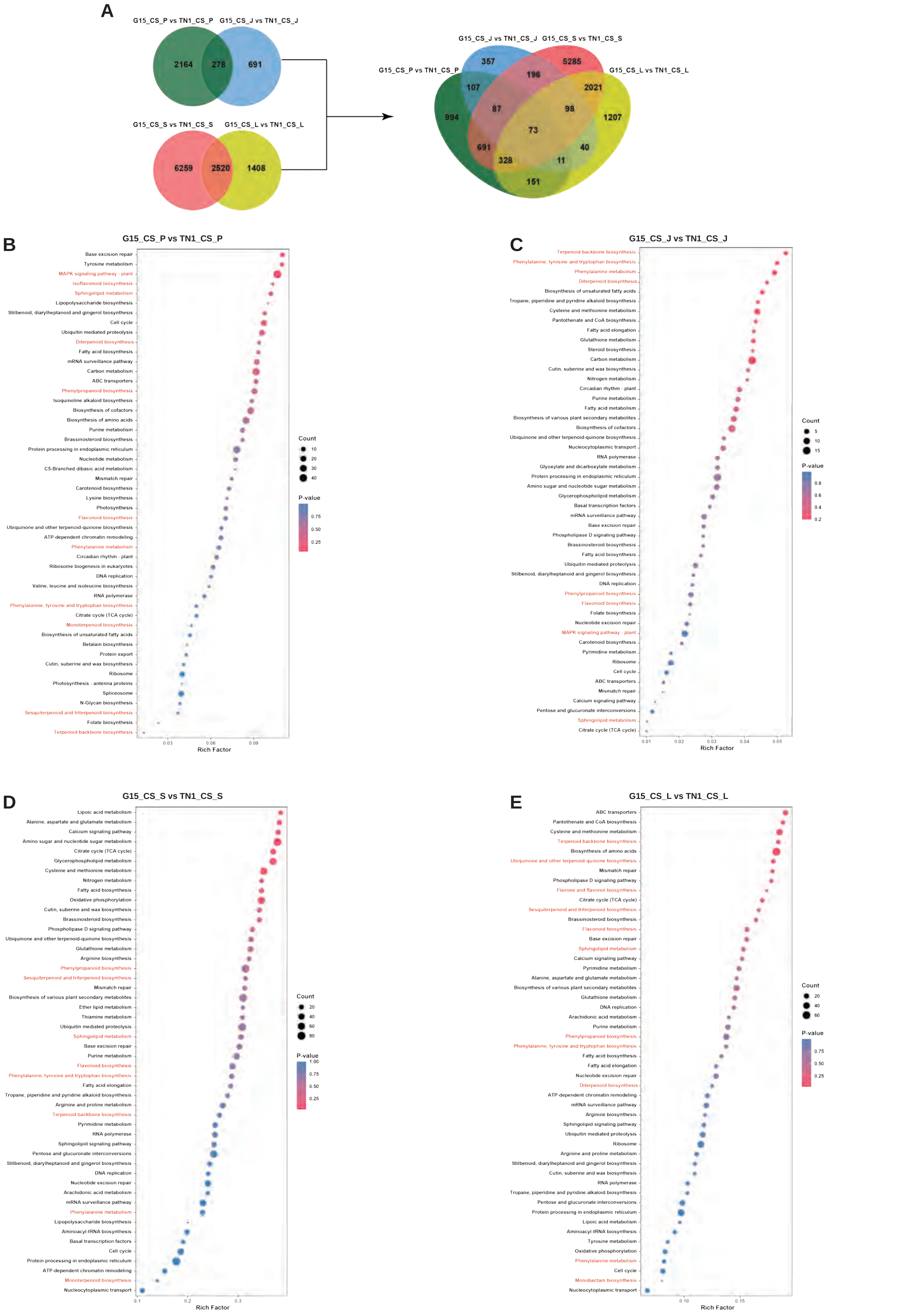

Supplementary Figure S12. The KEGG enrichment analysis of DEGs between G15 and TN1 passion fruits. A. Venn diagram analysis of common and specific genes between different pairwise comparisons. **B-E.** KEGG enrichment analysis of peels (P), pulp (J), stem (S) and leaf (L) of DEGs from G15 compared to TN1 varieties in cold treatment, respectively. Circle color denotes the P-value, circle size is proportional to the number of DAMs involved in the pathway (Count).

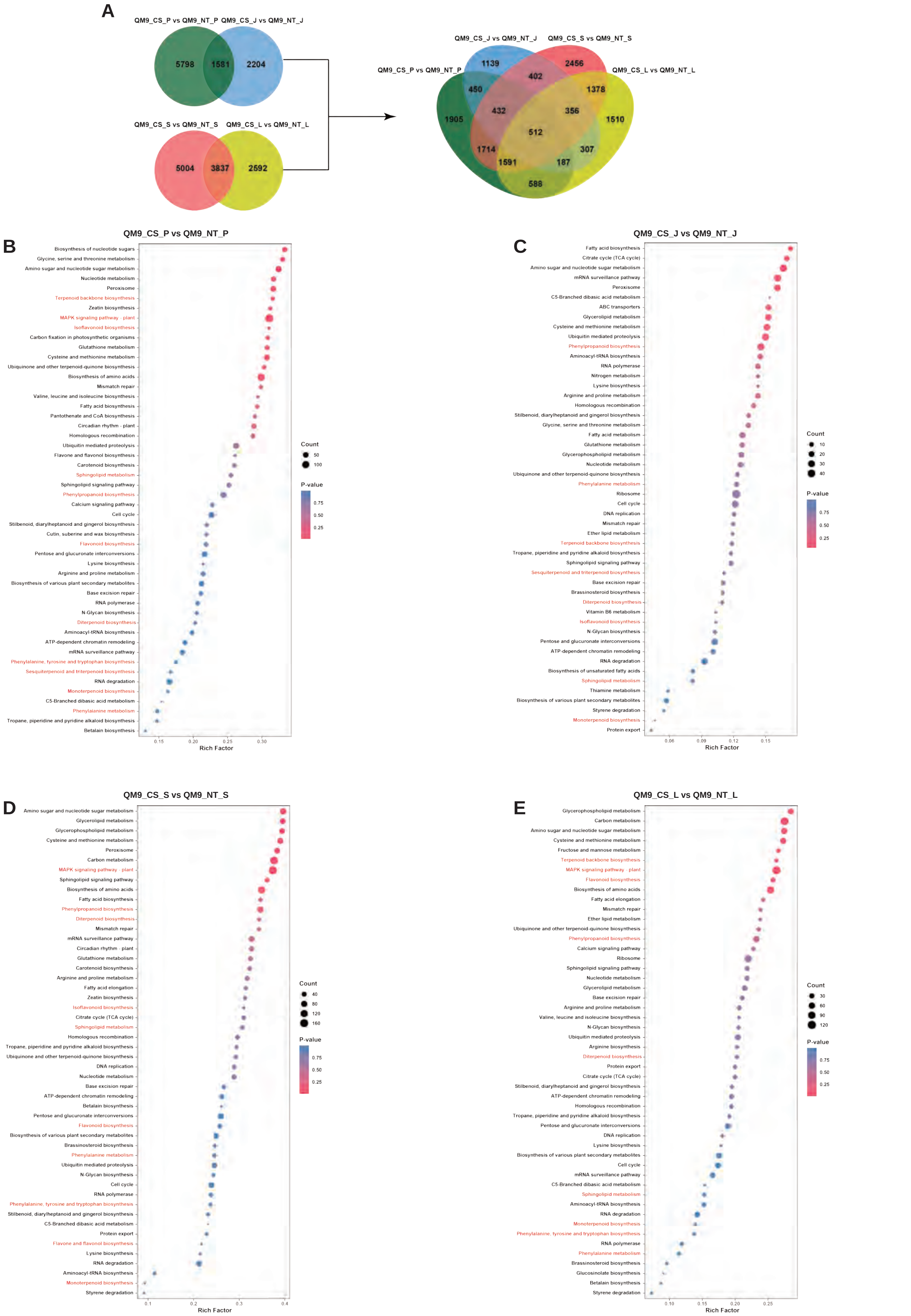

Supplementary Figure S13. The KEGG enrichment analysis of DEGs of cold-sensitive (QM9) passion fruit between cold stage and normal temperature. A. Venn diagram analysis of common and specific genes between different pairwise comparisons. **B-E.** KEGG enrichment analysis of peels (P), pulp (J), stem (S) and leaf (L) of DEGs between QM9_NT vs. QM9_CS, respectively. Circle color denotes the P-value, circle size is proportional to the number of DAMs involved in the pathway (Count).

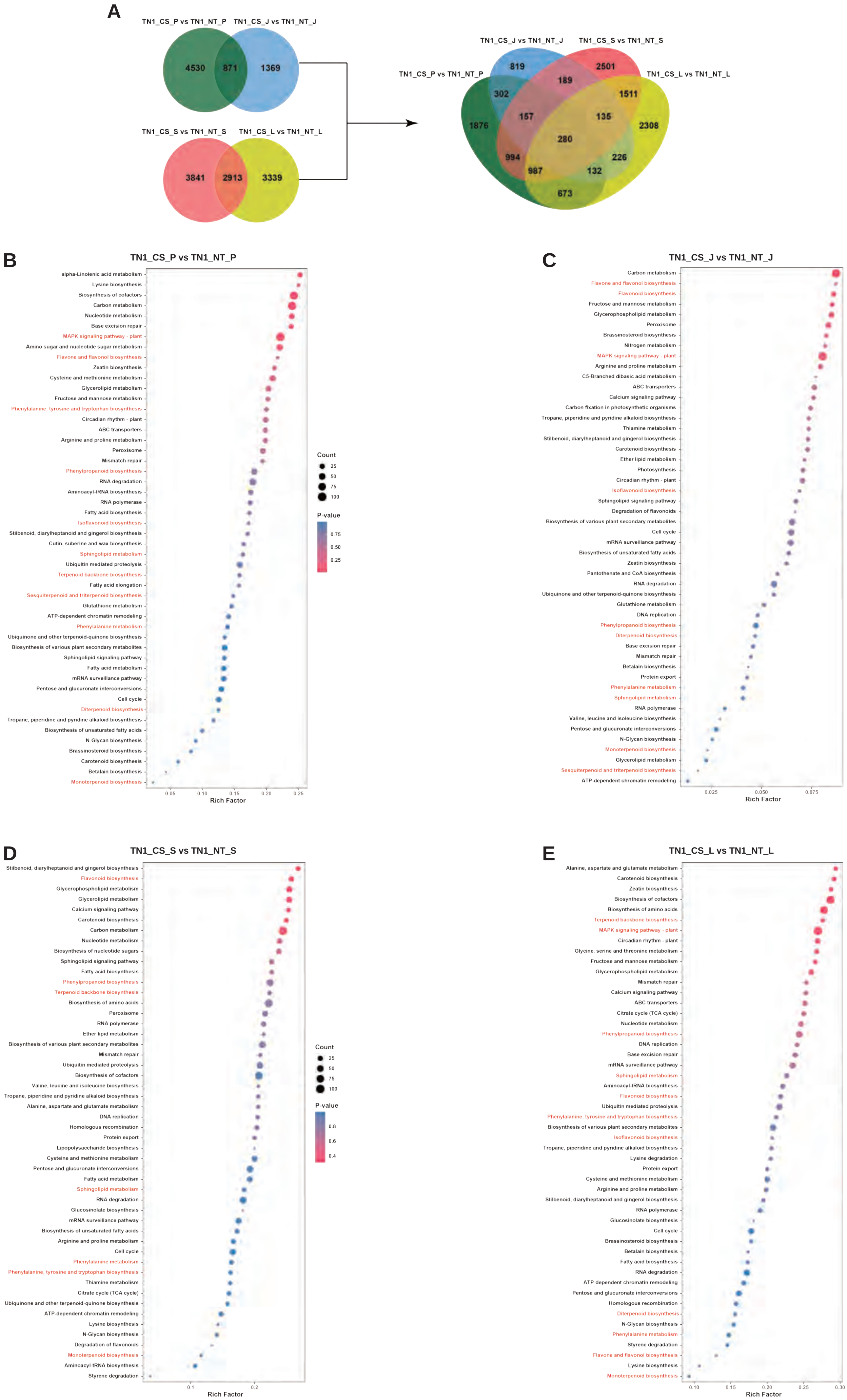

Supplementary Figure S13. The KEGG enrichment analysis of DEGs of cold-sensitive (TN1) passion fruit between cold stage and normal temperature. A. Venn diagram analysis of common and specific genes between different pairwise comparisons. **B-E.** KEGG enrichment analysis of peels (P), pulp (J), stem (S) and leaf (L) of DEGs between TN1_NT vs. TN1_CS, respectively. Circle color denotes the P-value, circle size is proportional to the number of DAMs involved in the pathway (Count).

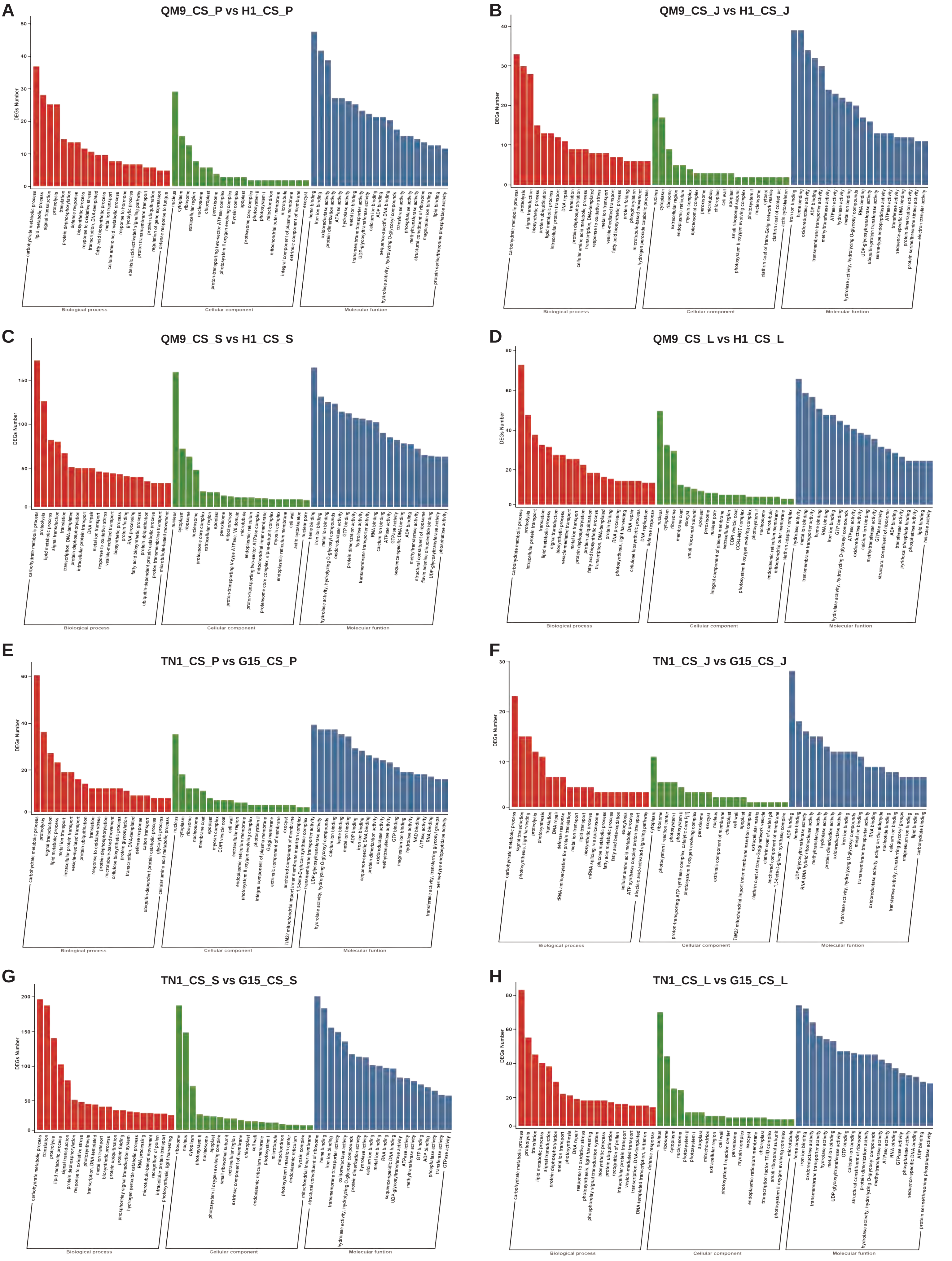

Supplementary Figure S15. The GO enrichment analysis of DEGs between cold-tolerant and cold-sensitive passion fruits. A-D. GO enrichment analysis of peels (P), pulp (J), stem (S) and leaf (L) of DEGs between QM9 vs. H1, respectively. **E-H.** GO enrichment analysis of P, J, S and L of DEGs between TN1 vs. G15, respectively.

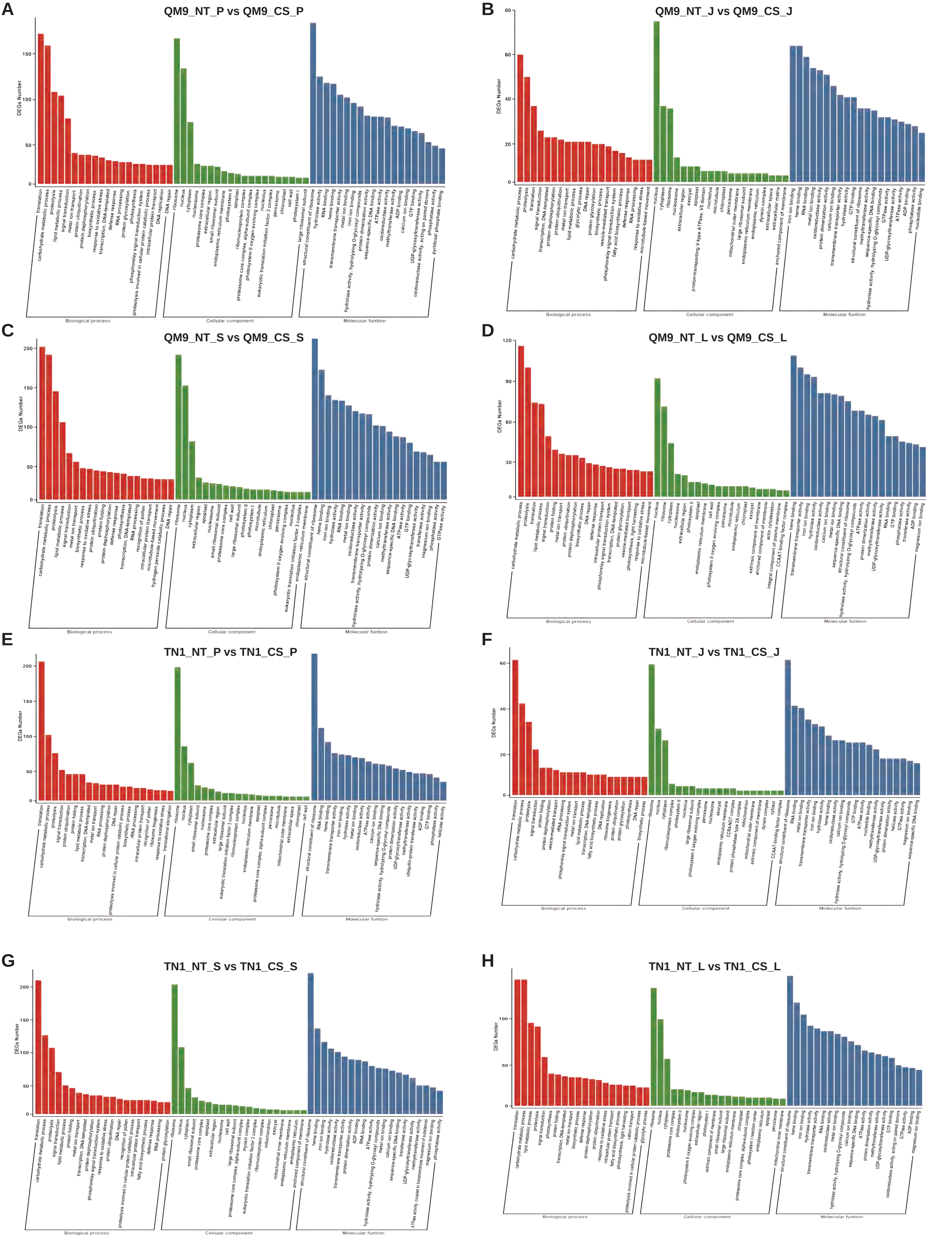

Supplementary Figure S16. The GO enrichment analysis of DEGs of cold-sensitive passion fruits between cold stage and normal temperature. A-D. GO enrichment analysis of peels (P), pulp (J), stem (S) and leaf (L) of DEGs between QM9_NT vs. QM9_CS, respectively. **E-H.** GO enrichment analysis of P, J, S and L of DEGs between TN1_NT vs. TN1_CS, respectively.

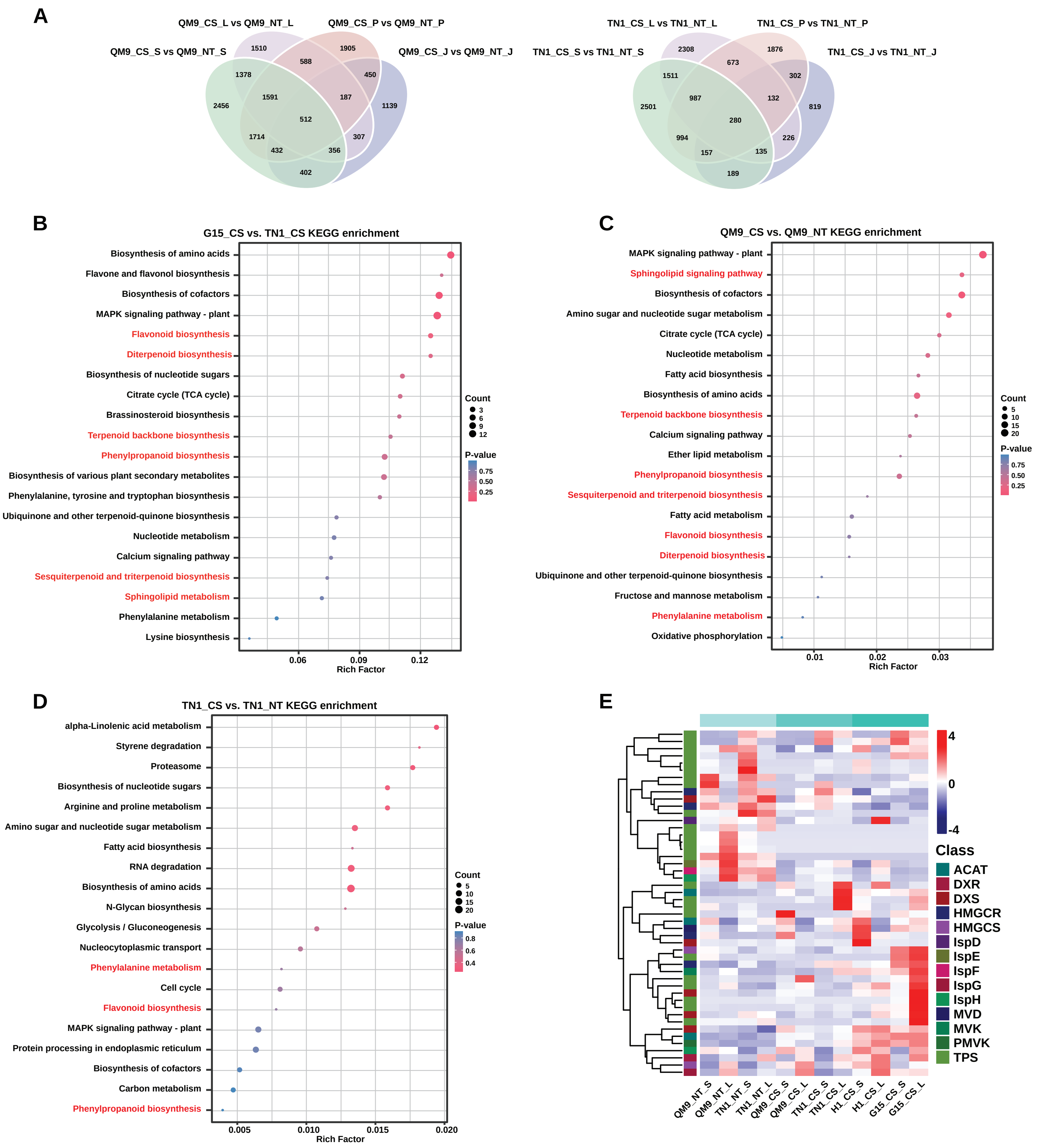

Supplemental Figure S17. Biosynthesis pathways identification of cold response. A. Venn diagram analysis of common and specific genes between different pairwise comparisons. **B, C** and **D.** The most enriched pathways among the species-dependent differentially expressed genes unique to G15_CS vs. TN1_CS (**B**), QM9_NT vs. QM9_CS (**C**), and TN1_NT vs. TN1_CS (**D**). **E.** Heatmap depicting gene expression in the biosynthesis pathway of terpenoids in the stem (S) and leaf (L) of various passion fruit varieties.

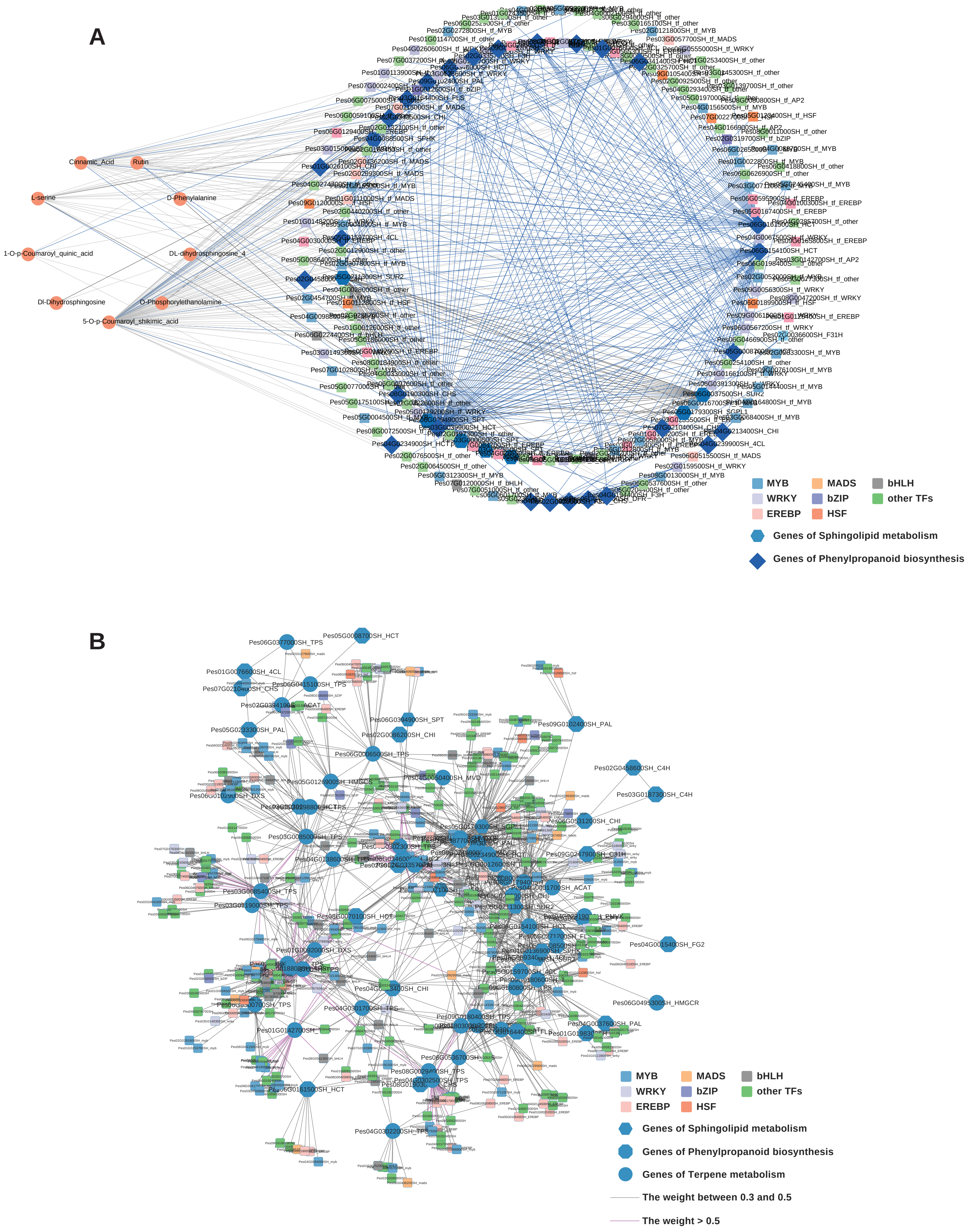

Supplementary Figure S18. Validation of cold resistance related genes. **A.** Correlation network highlighting the relationships between hub genes (including PesSPT, PesHCT) and key metabolic profiles. B. Transcriptional regulation network of genes in sphingolipid, phenylpropanoid, and terpene metabolism pathways. The squares with various colors depict separate transcription factor families that have a high correlation with these genes. Purple lines indicate correlations greater than 0.5, while grey lines represent correlations between 0.3 and 0.5.

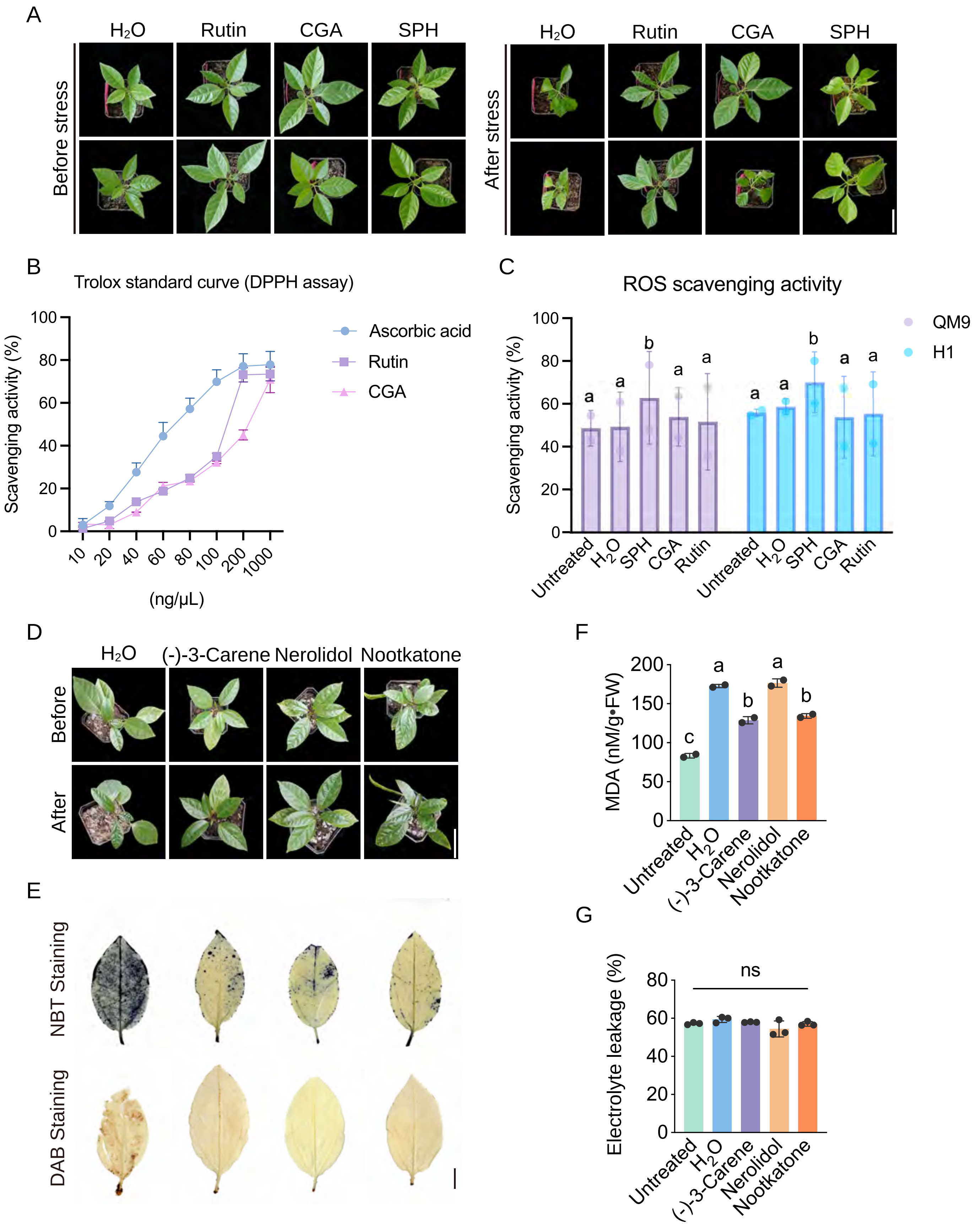

Supplementary Figure S19. Phenotypes of plants treated with exogenous compounds. **A**. Phenotypic of QM9 pretreated with sphingosine (SPH), chlorogenic acid (CGA), rutin and water before and after cold stress. Scale bar, 5 cm. **B,C**. Analysis of total antioxidant capacity in passion fruit after exogenous application of sphinganine, chlorogenic acid, and rutin. (**B**). Standard curves of DPPH radical scavenging activity for ascorbic acid, rutin and chlorogenic acid (CGA). (**C**). DPPH radical scavenging activity of passion fruit lines QM9 and H1 following exogenous application of sphinganine (SPH), chlorogenic acid, and rutin. **D.** Phenotypic of QM9 pretreated with (-)-3-carene, nerolidol, nootkatone and water before and after cold stress. Scale bar, 5 cm. **E**. DAB and NBT staining of pretreated QM9 leaves under cold treatment. Scale bar, 2 cm. **F,G**. Malondialdehyde (MDA) content (**F**) and electrolyte leakage (**G**) measured after cold treatment. Significant differences determined by two-way ANOVA in (**C**). Significant differences determined by one-way ANOVA in (**F**, **G**) (*P < 0.05, **P < 0.01, ***P < 0.001, ns: no significant difference). Data represent means ± SD (n = 3 in (**F**, **G**).

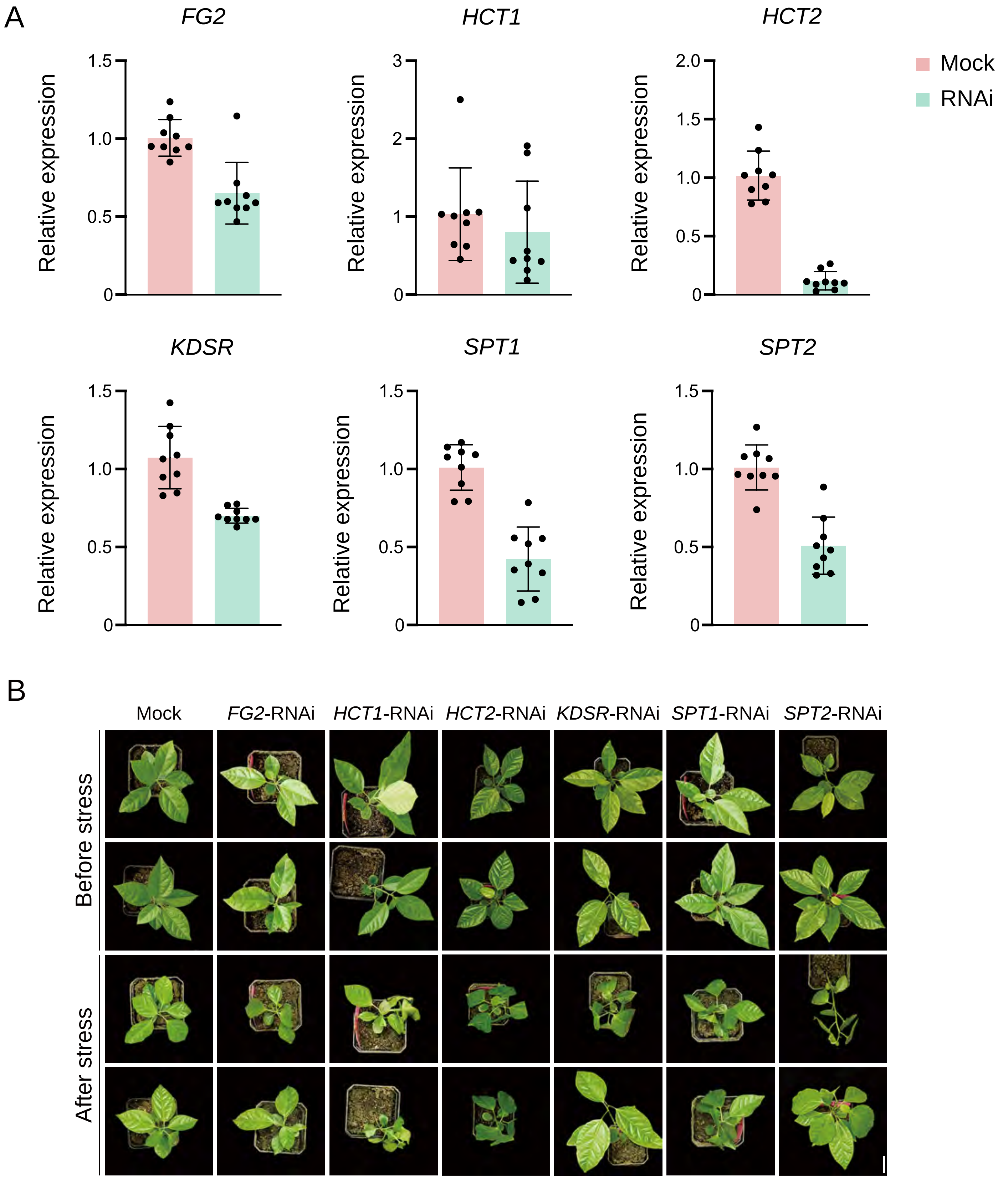

Supplementary Figure S20. Molecular identification and phenotypic characterization of VIGS plants. A. RT-qPCR was used to identification of the the target genes expression of VIGS plants. Mock, expressing empty TRV1 and TRV2. Error bars indicate ± SD (n = 3). **B.** Phenotypes of VIGS-line H1 (silencing cold-tolerance-related genes) and the mock control plants (empty, TRV1 + TRV2) before and after cold treatment. Scale bar, 5 cm.
